# Exactly solvable model for a velocity jump observed in crack propagation in viscoelastic solids

**DOI:** 10.1038/s41598-017-07214-8

**Published:** 2017-08-14

**Authors:** Naoyuki Sakumichi, Ko Okumura

**Affiliations:** 1 Soft Matter Center, Ochanomizu University, Bunkyo-ku, Tokyo 112-8610 Japan; 2 Department of Physics, Ochanomizu University, Bunkyo-ku, Tokyo 112-8610 Japan

## Abstract

Needs to impart appropriate elasticity and high toughness to viscoelastic polymer materials are ubiquitous in industries such as concerning automobiles and medical devices. One of the major problems to overcome for toughening is catastrophic failure linked to a velocity jump, i.e., a sharp transition in the velocity of crack propagation occurred in a narrow range of the applied load. However, its physical origin has remained an enigma despite previous studies over 60 years. Here, we propose an exactly solvable model that exhibits the velocity jump incorporating linear viscoelasticity with a cutoff length for a continuum description. With the exact solution, we elucidate the physical origin of the velocity jump: it emerges from a dynamic glass transition in the vicinity of the propagating crack tip. We further quantify the velocity jump together with slow- and fast-velocity regimes of crack propagation, which would stimulate the development of tough polymer materials.

## Introduction

Polymer-based viscoelastic materials are characterized by two elastic moduli *E*_0_ and *E*_∞_ corresponding to (soft) rubbery and (hard) glassy states, respectively^1, 2^. From this standard picture, one can understand generic features of the dependence of fracture energy on the velocity of crack propagation^3, 4^: the fracture energy *G* (twice the energy required to create a crack surface of unit area^5^) starts from a static value *G*_0_ and increases with the velocity *V* to the value *λG*_0_ with the ratio *λ* ≡ *E*_∞_/*E*_0_ (≃10^2^–10^3^)^6–8^. This is because strong dissipation occurs at places far from the crack tip, whereas *G*_0_ is well described by the cutting energy of chemical bonds and an effective cross-link distance^9^.

**Figure 1:**
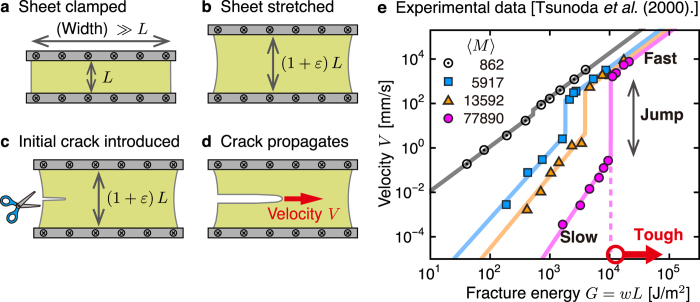
Velocity jump observed in the fixed-grip crack propagation. (**a**–**d**) Schematic illustrations of the fixed-grip crack propagation investigated in the present study. To achieve a constant-velocity crack propagation, we perform the following four steps: (**a**) we clamp the top and bottom edges of the sheet of height *L*; (**b**) we stretch the sheet to a fixed strain *ε*; (**c**) we introduce a small crack to initiate crack propagation; (**d**) after a short transient time, the crack propagates at a constant velocity *V* under the fixed strain *ε*. In the fixed-grip crack propagation, the fracture energy *G* and the energy release rate, which is expressed as *wL* under the fixed-grip condition, take the same value: *G* = *wL*. Here, *w* is the initially applied energy density. (**e**) Typical experimental results, *G* vs. *V*, obtained from the fixed-grip crack propagation by using elastomers filled with carbon black particles (taken from ref. 14). With increase in fracture energy, the slow-velocity regime (straight line on the low-velocity side) is terminated by an abrupt velocity jump, after which follows the fast-velocity regime (straight line on the high-velocity side). Here, 〈*M*〉 represent the average molar mass between nearest cross-links. In this series of experiments, a systematic increase in the cross-link distance leads to increase in the transition energy. Toughening is achieved by increasing the fracture energy at the transition point.

To further investigate dynamic properties of *G* as a function of *V*, crack propagation experiment performed under a fixed-grip (or pure-shear) condition possesses significant advantages. We illustrate this experiment in Fig. 1a–d: a long sheet of height *L* is subject to a fixed strain *ε* before and after the initiation of crack propagation, unlike other experiments based on peeling, tearing, cyclic loads, etc.^10, 11^. Advantages of the fixed-grip experiment are also stressed in ref. 12, and here we emphasize the following two points. (i) A steady-state crack propagation is realized with no work done by the external force, which leads to the equality *G* = *wL* ^10, 13^ with the initially applied elastic energy density

1 $$w(\varepsilon )\equiv {\int }_{0}^{\varepsilon }\sigma (\epsilon )d\epsilon ,$$

where *σ* is the stress. (ii) The experiment shown in Fig. 1e ^14^ and many other experiments^15–17^ indicate that the *G*-*V* plots exhibit an intriguing structure for elastomers: the velocity *V* jumps at a critical value *G* = *G*_*c*_, causing a transition from the slow-velocity ($V\lesssim 1$ mm/s) to fast-velocity ($V\gtrsim {10}^{3}$ mm/s) regime. This *G*-*V* structure reveals that toughness is achieved by increasing the critical value *G*_*c*_ because such an increase reduces the risk of a velocity jump, which can trigger catastrophic failure.

Theoretical understanding of the velocity jump has been very limited, although it is important for toughening polymer materials. Previous theories based on linear fracture mechanics^5^ and linear viscoelasticity^1^ are unable to reproduce the velocity jump^12, 18, 19^. Although there is a theory that reproduces the jump^20^, the theory predicts an extremely high-temperature region near the crack tip whereas only a slight temperature-increase was experimentally observed^21^.

In this article, we propose a minimal model that exhibits the velocity jump observed in the fixed-grip crack propagation, incorporating linear viscoelasticity with using the two elastic moduli *E*_0_ and *E*_∞_. This is performed with a spirit similar to the ones with which one of the authors constructed simple and useful models for biological composites^22–24^. From the proposed model, we obtain successfully an exact analytical relation between the initially applied energy density *w* and the crack propagation velocity *V*. As a result, we find simple expressions characterizing the transition point. These expressions provide guiding principles to reduce the risk of the jump, which can trigger catastrophic failure. Furthermore, we elucidate the physical mechanism that leads to the jump, indicating a direct link to dynamic emergence of a glassy state at the crack tip, and our results imply that the jump could be universally observed in a broad class of viscoelastic materials in addition to elastomers.

## Results

### Minimal model that exhibits the velocity jump

**Figure 2:**
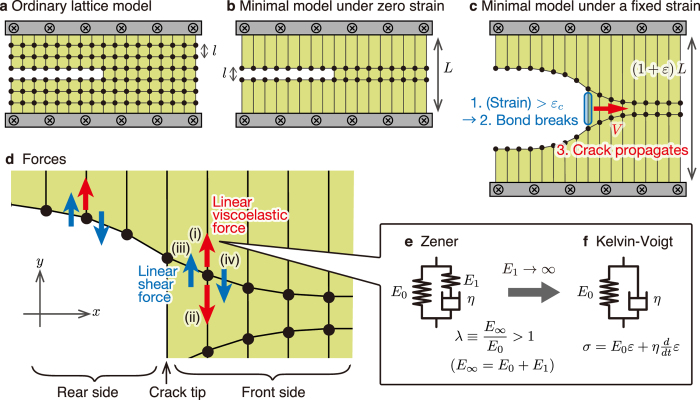
Minimal model for the straight-line crack propagation. (**a**) Two-dimensional square-lattice model of a sheet with a line crack. Here, *l* is the lattice spacing. Each lattice point interacts with the nearest-neighbor points. We introduce a line crack by cutting bonds, i.e., we set the interactions of the corresponding bonds to zero. (**b**) Minimal model obtained by coarse-graining the lattice in (**a**). We decimate all the lattice points except for the points on the two horizontal lines on which the two surfaces of the line crack are positioned, where *L* is the height of the sheet under zero strain. (**c**) Mechanism of the crack propagation. When the spring at the crack tip (encircled by a blue ellipse) is stretched to the critical strain *ε*_*c*_, the bond at the tip breaks, and, after a certain time, the next bond at the tip is stretched to *ε*_*c*_. This cycle continues during the crack propagation. (**d**) Forces acting on a lattice point. On each point in (**c**) located at the front side of the crack tip, four forces act: (i) one from the top boundary, (ii) one from the point below, and (iii, iv) the remaining two reflecting shear and acting from the left and right nearest-neighbor points. For each point located at the rear side, one force from the point below is missing. (**e**) Zener element. This element is a parallel connection of two components: a spring (elastic modulus *E*_0_) and a Maxwell element, i.e., a serial connection of another spring (modulus *E*_1_) and a dashpot (viscosity *η*). (**f**) Kelvin-Voigt element obtained from a Zener element in the large *E*_1_ limit, in which *λ* ≡ *E*_∞_/*E*_0_ → ∞.

To construct the minimal model, we start from the two-dimensional square-lattice model (Fig. 2a), often used to simulate the structure and dynamics of sheet materials, with the lattice spacing *l* and the sheet height *L* under zero strain. Then, we derive a simplified model illustrated in Fig. 2b by decimating most of the lattice points. As shown in Fig. 2c, the survivors (lattice points) represent the minimum number of variables essential to describe crack propagation. To realize a crack propagation in the *x*-direction (i.e., horizontal direction), we assume that each bond is broken if the local strain at the crack tip is larger than the critical strain *ε*_*c*_. For simplicity, we assume that the sheet is symmetric about the *x*-axis, and thus we consider only the lattice points on the upper side.

We explain the forces acting on each remaining lattice point on the upper side illustrated in Fig. 2d. We assume that Poisson’s ratio is zero. Thus, the forces always orient towards the *y*-direction (i.e., vertical direction) and each point can move only in the *y*-direction (see Supplementary Section I for details). Let *u*_*i*_ be the *y*-coordinate of the *i*-th point. The equation of motion of lattice points in the *y*-direction is given by

2 $$m\frac{{\partial }^{2}}{\partial {t}^{2}}{u}_{i}=K({u}_{i}-{u}_{i+1}+{u}_{i}-{u}_{i-1})+{F}_{i},$$

where $K({u}_{i}-{u}_{i+1}+{u}_{i}-{u}_{i-1})$ represents linear-elastic shear force acting from the left and right nearest-neighbor points, and *F*_*i*_ represents viscoelastic tensile force acting from the top boundary and the point below. The tensile force *F*_*i*_ is described by a Zener element in Fig. 2e characterized by two elastic moduli (*E*_0_ and *E*_∞_) and viscous dissipation (*η*), as in de Gennes’ trumpet model^6–8^. As illustrated in Fig. 2d, *F*_*i*_ takes two different forms, depending on whether the *i*-th lattice point is located on the rear (i.e., left) or front (i.e., right) side of the crack tip because one of the four forces is missing on the rear side. We relegate the explicit form of *F*_*i*_ to Supplementary Section III to avoid complication. Instead, we give the explicit form of *F*_*i*_ in the limit, *E*_∞_ → ∞, in which a Zener element reduces to a Kelvin-Voigt element (Fig. 2f). In this limit, the tensile forces on the rear and front sides take the following form:

3 $${F}_{i}=\{\begin{array}{cc}\alpha [c-l({E}_{0}{u}_{i}+\eta \frac{{\rm{\partial }}}{{\rm{\partial }}t}{u}_{i})] & {\rm{r}}{\rm{e}}{\rm{a}}{\rm{r}}\,{\rm{s}}{\rm{i}}{\rm{d}}{\rm{e}}\\ \alpha [c-L({E}_{0}{u}_{i}+\eta \frac{{\rm{\partial }}}{{\rm{\partial }}t}{u}_{i})] & {\rm{f}}{\rm{r}}{\rm{o}}{\rm{n}}{\rm{t}}\,{\rm{s}}{\rm{i}}{\rm{d}}{\rm{e}},\end{array}$$

where *α* and *c* are constants.

**Figure 3:**
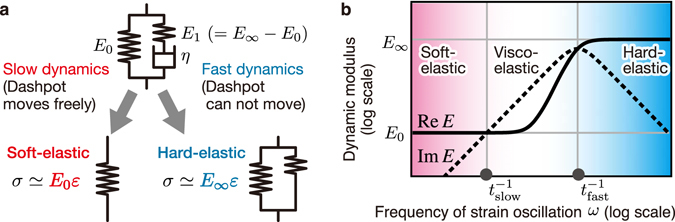
Three types of dynamic responses of a Zener element: soft-elastic, viscoelastic, and hard-elastic responses. (**a**) Dynamical response of a Zener element to adequately slow or fast stretch (see, text). (**b**) Dynamic modulus *E*(*ω*) = Re*E*(*ω*) + *i*Im*E*(*ω*) as a function of an angular frequency of strain oscillation *ω* in a Zener element. Here, Re*E*(*ω*) and Im*E*(*ω*) are the storage and loss moduli, respectively. We plot *E*(*ω*) = *E*_0_(1 + *iωt*_slow_)/(1 + *iωt*_fast_) obtained from the stress-strain relation in equation (4), with ${t}_{{\rm{fast}}}\simeq \eta /{E}_{\infty }$ and ${t}_{{\rm{slow}}}\simeq \eta /{E}_{0}$. In the (rubbery) soft-elastic and (glassy) hard-elastic regimes, the dynamics are elastic and characterized by *E*_0_ and *E*_∞_, respectively, whereas in the viscoelastic regime the dynamics is governed by *η*, *E*_0_, and *E*_∞_.

A Zener element^25–27^ is one of the simplest models to represent typical viscoelastic behavior around a glass transition for polymer materials. As illustrated in Fig. 3a, when stretched with an adequately slow speed, a Zener element exhibits (rubbery) soft-elastic behavior, because the dashpot moves freely without any friction: the elastic modulus is small and approximately given by *E*_0_. On the other hand, when stretched with an adequately fast speed, a Zener element exhibits (glassy) hard-elastic behavior, because the dashpot does not have enough time to move: the elastic modulus is large and approximately given by *E*_∞_. For a conventional elastomer, $\lambda \equiv {E}_{\infty }/{E}_{0}\simeq {10}^{3}$. The relation between stress (*σ*) and strain (${\mathcal E}$) of Zener element is given by

4 $$(1+{t}_{{\rm{fast}}}\frac{d}{dt})\sigma (t)=(1+{t}_{{\rm{slow}}}\frac{d}{dt}){E}_{0} {\mathcal E} (t),$$

with ${t}_{{\rm{fast}}}\equiv \eta /{E}_{1}\simeq \eta /{E}_{\infty }$ and ${t}_{{\rm{slow}}}\equiv \eta /{E}_{0}+\eta /{E}_{1}\simeq \eta /{E}_{0}$. As shown in Fig. 3b, equation (4) gives a dynamic modulus (i.e., the ratio of stress to strain under oscillatory conditions), mimicking a typical viscoelastic behavior around a glass transition for polymer materials.

### Exact analytical relation between *w* and *V*

The minimal model allows us to derive an exact analytical relationship between *w* = *G*/*L* and *V*, in a continuum limit in the *x*-direction, in which we replace *u*_*i*_ − *u*_*i*+1_ + *u*_*i*_ − *u*_*i*−1_ with *l* ^2^∂^2^ *u*(*x*, *t*)/∂*x* ^2^ in equation (2). For simplicity, we further take the overdamped (i.e., inertialess) limit, i.e., we neglect the inertial term *m*∂^2^ *u*/∂*t* ^2^. The latter limit is valid if the crack propagation velocity under question is much smaller than the shear wave velocity $l\sqrt{K/m}$. Under the two limits, we rewrite equation (2) as

5 $$0={l}^{2}K\frac{{\partial }^{2}}{\partial {x}^{2}}u(x,t)+F(x,t\mathrm{)}.$$

Here, the form of *F*(*x*, *t*) changes depending on whether the position *x* is located on the rear or front side of the crack tip as implied above, and equation (5) satisfies appropriate boundary conditions at *x* = ±∞ and matching conditions at the crack tip.

We now explain the main result: an exact analytical relation between *w* and *V* (see Methods for the derivation). Since the present model is initially (i.e., before the crack propagates) at rest with a fixed *ε* without shear, it behaves as a linear elastic material governed by *σ* = *E*_0_ *ε* and the initially applied energy density is given by *w* = *E*_0_ *ε* ^2^/2. Let *N* ≡ *L*/*l* be the dimensionless parameter of the length scale in the *y*-direction. For $\varepsilon \le {\varepsilon }_{c}/\sqrt{N}$ the crack does not propagate (*V* = 0) and for $\varepsilon \ge {\varepsilon }_{c}\lambda /(\sqrt{N}+\lambda -1)$ any constant-velocity solutions do not exist. (When $\varepsilon \to {\varepsilon }_{c}\lambda /(\sqrt{N}+\lambda -1)$, the velocity *V* diverges to infinity, which is an artifact resulting from the overdamped limit). The crack propagates with a constant velocity only in the range ${\varepsilon }_{c}/\sqrt{N} < \varepsilon  < {\varepsilon }_{c}\lambda /(\sqrt{N}+\lambda -1)$, or equivalently, in the range *w*_0_ < *w* < *w*_∞_. Here, ${w}_{0}(\equiv {E}_{0}{\varepsilon }_{c}^{2}\mathrm{/(2}N))$ and *w*_∞_ are the minimum and maximum values of *w* for the propagation with a constant velocity, respectively. In this range, the relation between *w* and *V* is given by

6 $$\frac{w}{{w}_{0}}=N{[\frac{\frac{1}{\lambda -1}(\frac{N}{{\xi }_{1}{\xi }_{N}}+\lambda )\frac{V}{{V}_{0}}+{\xi }_{1}+{\xi }_{N}}{\frac{1}{\lambda -1}(N+\frac{N}{{\xi }_{1}{\xi }_{N}}+\lambda -1)\frac{V}{{V}_{0}}+N{\xi }_{1}+{\xi }_{N}}]}^{2},$$

with a reference velocity ${V}_{0}\equiv \frac{l}{\eta }\sqrt{\frac{1}{2}(1-\frac{1}{N}){E}_{0}\mu }$, where *μ* is an effective shear modulus. We note that *V*_0_ scales as *l*/*t*_0_ with the (largest) relaxation time *t*_0_ ≡ *η*/*E*_0_, in practical cases with $l\ll L$, in which *μ* scales as *E*_0_. In equation (6), the dimensionless length scale *ξ*_*N*_ is the positive real solution of the following cubic equation for *ξ*:

7 $${\xi }^{3}+\frac{\lambda V}{(\lambda -1){V}_{0}}{\xi }^{2}-N\xi -\frac{NV}{(\lambda -1){V}_{0}}=0,$$

which has a unique positive real solution as guaranteed by Lemma 1 in Supplementary Section III-B. The explicit form of *ξ*_*N*_ is given by Cardano’s formula^28^ for the solution of a cubic equation. We obtain *ξ*_1_ by substituting *N* = 1 to *ξ*_*N*_.

**Figure 4:**
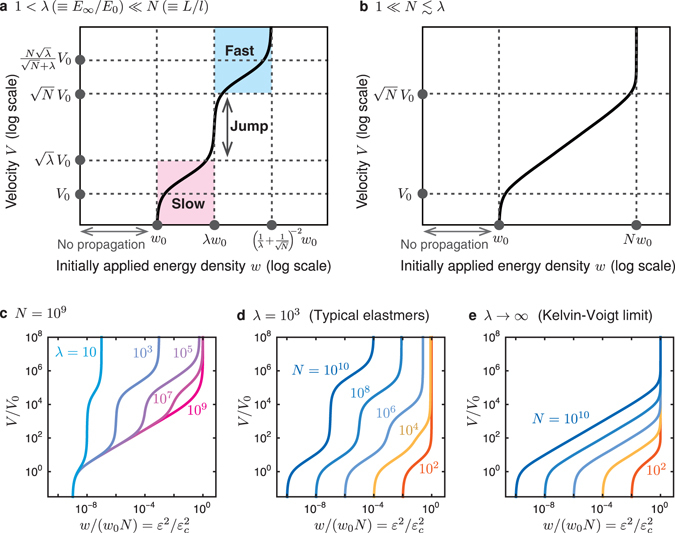
Reproduced velocity jump and simple characterization of the *w*-*V* curve. (**a**,**b**) Two representative plots of the crack propagation velocity *V* as a function of the initially applied energy density *w*. The cases (**a**) with and (**b**) without velocity jump are obtained for $1 < \lambda \ll N$ and $1\ll N\lesssim \lambda$, respectively. (These plots are obtained for (**a**) *λ* = 10^3^, *N* = 10^9^ and (**b**) *λ* → ∞, *N* = 10^9^). Four characteristic velocity-scales and three energy-scales are indicated in (a), which are important for toughening. (**c**–**e**) *V*/*V*_0_ vs. $w/({w}_{0}N)={\varepsilon }^{2}/{\varepsilon }_{c}^{2}$, obtained from equation (1) on a log-log scale. The normalization factors for velocity and energy are ${V}_{0}\simeq l{E}_{0}/\eta$ and ${w}_{0}\equiv {E}_{0}l{\varepsilon }_{c}^{2}/(2L)$, respectively. The cases with velocity jump are demonstrated for various *λ* with a fixed *N* in (**c**) and for various *N* with a fixed *λ* in (**d**). The Kelvin-Voigt limit, *λ* → ∞, is shown for various *N* in (**e**) as an example of the case without velocity jump.

As illustrated in Fig. 4a, equation (6) guarantees the existence of the velocity jump for $\lambda \ll N\equiv L/l$. The existence condition $\lambda \ll N$ is derived in Supplementary Section IV-B (see, Theorem 3) and is well satisfied in conventional elastomers for regular specimen sizes ($\lambda \simeq {10}^{3}$, $L\simeq 10$ cm, and $l\simeq 10$ nm). Since a Zener element generally represents typical viscoelastic behavior around a glass transition, the present model is relevant to a broad class of materials beyond elastomers: the velocity jump is expected to be a universal phenomenon in polymer materials such as gels and resins. Note that the present model does not reproduce the velocity jump for $N\lesssim \lambda$ (including the Kelvin-Voigt limit, *λ* → ∞) as illustrated in Fig. 4b. Figure 4c–e demonstrates how equation (6) depends on *λ* and *N*.

### Guiding principles to develop tough polymer materials

The exact relation in equation (6) leads to simple expressions for the four points characterizing the *w* − *V* curve given in Fig. 4a, such as (*w*_0_, *V*_0_) and $(\lambda {w}_{0},\sqrt{\lambda }{V}_{0})$. In particular, the point $(\lambda {w}_{0},\sqrt{\lambda }{V}_{0})$ shows that the velocity jump occurs at *w* = *w*_jump_, where

8 $${w}_{{\rm{jump}}}\equiv \lambda {w}_{0}=\frac{l{E}_{\infty }{\varepsilon }_{c}^{2}}{2L}.$$

The transition energy density *w*_jump_ given in equation (8) is consistent with empirical knowledge in polymer science. For instance, Fig. 1e experimentally shows that the transition energy *G*_*c*_ = *w*_jump_ *L* increases as the cross-link distance (i.e., the parameter *l*) increases^14^. This feature is consistent with equation (8) because *E*_∞_ and *ε*_*c*_ are approximately constant even for different 〈*M*〉 in Fig. 1e (see, e.g., ref. 17).

Equation (8) gives the following guiding principles to develop tough polymer materials (i.e., to reduce the risk of a velocity jump, which can trigger catastrophic failure): the transition energy density *w*_jump_ is enhanced with increase in (i) the modulus *E*_∞_ of the glassy state and/or (ii) the lattice spacing *l*. Here, we can regard *l* as a characteristic length scale below which the continuum description is no longer valid: *l* is the largest length scale among scales such as the cross-link distance, the size of filler particles, the filler-particle distance, and the length scale of possible inhomogeneous structures in the sample. Equation (8) indicates that we can keep the appropriate principal elasticity *E*_0_ to develop tough polymer materials in principle, which is a practical advantage.

We here remark on the two sharp changes at *w* = *w*_0_ and $w={(\frac{1}{\lambda }+\frac{1}{\sqrt{N}})}^{-2}{w}_{0}$ in Fig. 4a. The former results from a fundamental property of the log-log plot: *w* linearly approaches a constant value *w*_0_ as *V* approaches zero. (See equation (16) in Methods). As for the latter, *V* diverges to infinity as *w* approaches ${(\frac{1}{\lambda }+\frac{1}{\sqrt{N}})}^{-2}{w}_{0}$ (see equation (17) in Methods). However, as already mentioned, this divergence of *V* is an artifact coming from the overdamped limit, in which we neglect the inertial term in our governing equation. If we added the inertial term, the divergence would be suppressed.

### Physical origin of the velocity jump

To elucidate the physical origin of the velocity jump, we focus on a crossover among the three types of dynamic responses of Zener elements, corresponding to soft-elastic, viscoelastic, and hard-elastic regimes (Fig. 3b), depending on the time scale of the propagation dynamics. Since we are interested in a crack propagation closely related to relaxation responses (rather than oscillatory responses in Fig. 3b) of Zener elements, we introduce the two parameters

$${{\rm{\Psi }}}_{{\rm{soft}}}\equiv \frac{ {\mathcal E} }{{t}_{{\rm{slow}}}\frac{d}{dt} {\mathcal E} }\quad {\rm{and}}\quad {{\rm{\Psi }}}_{{\rm{hard}}}\equiv \frac{{t}_{{\rm{fast}}}\frac{d}{dt}\sigma }{\sigma },$$

to characterize the dynamic responses behind equation (4): (i) when ${{\rm{\Psi }}}_{{\rm{soft}}}\gg 1$ (and ${{\rm{\Psi }}}_{{\rm{hard}}}\ll 1$), equation (4) reduces to $\sigma ={E}_{0} {\mathcal E}$, which corresponds to the soft-elastic regime; (ii) when ${{\rm{\Psi }}}_{{\rm{hard}}}\gg 1$ (and ${{\rm{\Psi }}}_{{\rm{soft}}}\ll 1$), equation (4) reduces to $\sigma ={E}_{\infty } {\mathcal E}$ (with omission of an integral constant), which corresponds to the hard-elastic regime; (iii) when ${{\rm{\Psi }}}_{{\rm{soft}}}\lesssim 1$ and ${{\rm{\Psi }}}_{{\rm{hard}}}\lesssim 1$, viscous dissipation terms in equation (4) play a role in the dynamics, which corresponds to the viscoelastic regime.

**Figure 5:**
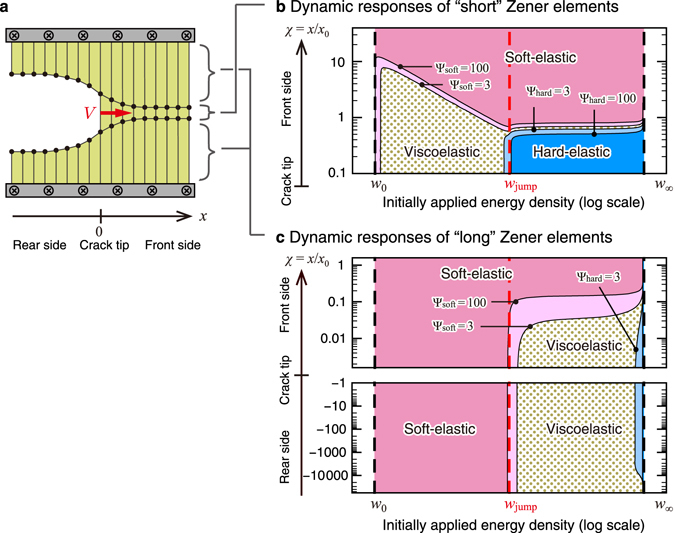
Dynamic responses of Zener elements. (**a**) “Short” and “long” Zener elements of natural length *l* and (*L* − *l*)/2, respectively. Here, we set the origin of the *x*-coordinate at the crack tip. (**b**,**c**) Representative behavior of dynamic responses of Zener elements for conventional elastomers (*λ* = 10^3^ and *N* = 10^9^). We show relaxational responses of Zener elements during a constant-velocity crack propagation, in contrast to vibrational responses in Fig. 3. The four curves are contours for Ψ_soft_ = 100, 3 and Ψ_hard_ = 3, 100 as a function of the initially applied energy density *w* and the distance from the crack tip *χ* ≡ *x*/*x*_0_. The conditions ${{\rm{\Psi }}}_{{\rm{soft}}}\gg 1$ and ${{\rm{\Psi }}}_{{\rm{hard}}}\gg 1$ correspond to soft- and hard-elastic regimes, respectively (see the text for details). Explicit forms of Ψ_soft_, Ψ_hard_, and *x*_0_ are given in Methods. Red dashed lines correspond to the velocity jump (*w*_jump_) and black dashed lines correspond to the minimum (*w*_0_) and maximum (*w*_∞_) values of *w* for with constant-velocity propagation.

By using the parameters Ψ_soft_ and Ψ_hard_, we show in Fig. 5 dynamic responses of the “short” and “long” Zener elements (see Fig. 5a) in the present model. To clarify physical pictures for the slow-velocity (*w*_0_ < *w* < *w*_jump_) and fast-velocity (*w*_jump_ < *w* < *w*_∞_) crack propagations and the velocity jump (*w* = *w*_jump_), we should pay attention to the moving Zener elements near the crack tip. In other words, the Zener elements far from the crack tip are almost in equilibrium and do not affect crack-propagation dynamics. For example, soft-elastic regimes in Fig. 5b,c are almost in equilibrium and play a minor role for crack propagations. Thus, we now focus on the viscoelastic and hard-elastic regimes in Fig. 5b,c. Figure 5b shows that the “short” Zener element in the vicinity of the crack tip is viscoelastic in the slow-velocity propagation (*w*_0_ < *w* < *w*_jump_) but is hard-elastic in the fast-velocity propagation (*w*_jump_ < *w* < *w*_∞_), with an abrupt change at the velocity jump (*w* = *w*_jump_). Figure 5c shows that the “long” Zener elements near the crack tip are soft-elastic and viscoelastic in slow- and fast-velocity propagations, respectively, with an abrupt change at *w* = *w*_jump_. Note that the viscoelastic regime far from the crack tip on the rear side ($-\chi \gg 1$) in Fig. 5c is almost in equilibrium and accompanied by exponentially-small viscous dissipation. In fact, the stress (*σ*), strain (${\mathcal E}$), and their time derivatives (given by equation (23) in Method) decay with the same exponential factor as the distance from the crack tip is increased, whereas Ψ_soft_ and Ψ_hard_, by definition, take finite values even at far distances.

**Figure 6:**
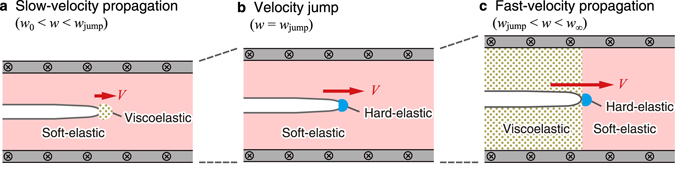
Physical pictures on the crack propagation revealed by the present exact solution. We draw the three illustrations based on Fig. 5. (**a**) The slow-velocity propagation is characterized by viscous dissipation in the vicinity of the crack tip. (**b**) The velocity jump induced by emergence of a hard-elastic regime (as a result of a dynamic glass transition) in the close vicinity of the crack tip. (**c**) The fast-velocity propagation is characterized by viscous dissipation on the rear side (with the hard-elastic regime in the close vicinity of the crack tip). Note that away from the crack tip viscous dissipation in the viscoelastic regime decays with the distance from the tip (see the text for details).

From the above observations, we can draw physical pictures for the slow- and fast-velocity crack propagations and the velocity jump as illustrated in Fig. 6: (i) the slow-velocity and fast-velocity crack propagations are characterized by viscous dissipation in the vicinity of the crack tip (Fig. 5b) and on the rear side (Fig. 5c), respectively, as illustrated in Fig. 6a and c; (ii) The velocity jump starts with the emergence of a hard-elastic regime near ahead of the crack tip (Fig. 5b) and ends with the emergence of a viscoelastic regime on the rear side (Fig. 5c), as illustrated in Fig. 6b. Since the appearance of a hard-elastic regime is a sign of the dynamic glass transition, we can interpret the onset of the velocity jump at *w* = *w*_jump_ (Fig. 6b) as the dynamic glass transition at the crack tip. Note that the glass transition occurs practically only in the close vicinity of the crack tip because the transition requires a strong stretch and such a stretch can occur only for short elements. This fact implies that a glass transition is easy to occur in crack propagation, and thus, we expect that even materials such as gels, in which glass transitions are difficult to occur, could exhibit a velocity jump.

## Discussion

In summary, we have proposed a minimal model that exhibits the velocity jump in viscoelastic solids for which an exact analytical solution is available. The exact relation given in equation (6) allows us to characterize the transition point as in equation (8) and such a simple expression is useful as guiding principles to develop tough polymer materials. In addition, we have elucidated the physical origin of the velocity jump as a dynamic glass transition in the vicinity of the propagating crack tip (see, Figs 5 and 6). Our result implies that the discontinuous transition in the crack propagation velocity is a universal phenomenon that could be observed in a broad class of viscoelastic materials.

The present results are useful both from practical and fundamental viewpoints. (i) Conventionally the development of new materials tends to be achieved by trials and errors; however, the expressions characterizing the marked points on the curve in Fig. 4a are simple enough to remove such trials and errors, and pave the way for a more efficient development of tough polymer materials. (ii) The minimal model proposed in this article is not restricted to the fixed-grip geometry; we can easily handle other types of crack experiments in the present framework by considering the time dependence of applied strain *ε*. For example, tensile and cyclic experiments are treated by setting *ε*(*t*) = *vt* and *ε*(*t*) = *A* sin (*ωt*), respectively. Here, *v* is the tensile velocity, and *A* and *ω* are the amplitude and the angular frequency of the oscillation. We will study this line of research elsewhere. (iii) The present results involve an interesting analogy to conventional phase transitions. There appear two quantities *ξ*_*N*_ and *ξ*_1_ associated with the front and rear sides, respectively, that play a role for the order parameter of the velocity jump in a sense that it changes form one characteristic value to the other as a function of an external control parameter (see Supplementary Fig. S4c). (iv) Connection to reaction-diffusion systems is an important issue to be explored. Equation (9) in Methods for Kelvin-Voigt limit (*λ* → ∞) belongs to the class of reaction-diffusion equation, $\frac{\partial }{\partial t}u=D\frac{{\partial }^{2}}{\partial {x}^{2}}u+R[u]$, and the counterpart for arbitrary *λ* forms a generalized class. Accordingly, the present generalization could enrich physical scenarios in reaction-diffusion systems in different disciplines, e.g., pattern formation in chemical reaction systems and morphogenesis in biology. In return, crack problems in viscoelastic materials can benefit from the field of reaction-diffusion systems. The present crack problem corresponds to a linear reaction term *R*[*u*] ∝ *u*, and nonlinear extension (e.g., Ramberg-Osgood stress-strain relation) is important for dealing with more practical materials. Such an extension could be solved with the aid of the accumulated mathematical knowledge in a well-developed field of reaction-diffusion systems^29^.

After completion of the present analytical work, experimental^30^ and numerical^31^ studies on the velocity jump were published. First, we compare the present study with the experimental study^30^. Figure 3d in ref. 30 suggests that the *G*-*V* plots, which exhibit the velocity jump, do not change when the specimen thickness is changed in the range 0.7–2.0 mm. This independence from thickness supports our two-dimensional modeling. Figure 12a in ref. 30 shows that *w*_jump_ is approximately proportional to the “fracture toughness” *w*_*c*_, when experiments were carried out with changing silica-filler content, cross-linker concentration, and temperature. Here, *w*_*c*_ is obtained from the area defined on the stress-strain curve: ${w}_{c}\equiv w({\varepsilon }_{c})={\int }_{0}^{{\varepsilon }_{c}}\sigma (\epsilon )d\epsilon$. (See equation (1)). Although *w*_*c*_ is calculated from a non-linear stress-strain curve in experiments, *w*_*c*_ can also be calculated in our linear model, in which *σ*(*ε*) = *E*_0_ *ε* and ${w}_{c}={E}_{0}{\varepsilon }_{c}^{2}\mathrm{/2}$. Thus, in our model, equation (8) is rewritten as *w*_jump_ = *w*_*c*_ *λl*/*L*, i.e., *w*_jump_ is proportional to *w*_*c*_. This feature is consistent with Figure 12a in ref. 30. Other results in ref. 30 are based on nonlinear elasticity and cannot be directly compared with ours. Second, we compare our analytical study with the numerical study^31^, which qualitatively reproduces the velocity jump by using a finite-element-method (FEM). Their numerical model takes into account nonlinear viscoelasticity introducing 30 material parameters, by quantitatively fitting the result of the experiment in ref. 17. Although they qualitatively reproduced jumps, their simulation result of the *G*-*V* plot shown in Fig. 1 in ref. 31 is not in quantitative agreement with the corresponding *G*-*V* plot in ref. 17. This discrepancy may be because of the finiteness of elements, which causes problems especially in the vicinity of the crack tip. In their study, they have not clarified the following two fundamental points: (i) whether nonlinear elasticity is necessary for the velocity jump; (ii) the relationship between the velocity jump and glass transition of the materials. Unlike their complicated numerical model, we have considered a minimal model based on linear viscoelasticity with only three material parameters (*E*_0_, *E*_∞_, and *η*), aiming at the elucidation of the physics of the velocity jump in a simple and clear manner. As a result, we have solved the model exactly and clarified the existence condition of the velocity jump and the relationship between the velocity jump and glass transition.

## Methods

### Derivation of the relation between *w* and *V*

To explain how to derive the exact relation between *w* and *V* given in equation (6), we first consider a more simplified model consisting of Kelvin-Voigt elements illustrated in Fig. 2f. This simpler model is obtained from the present model in the limit *λ* → ∞. Although this simpler model does not reproduce the velocity jump (see Fig. 4b and e), it is useful to understand the mathematical structure of the present model.

In this simpler model, the equation of motion of lattice points in the *y*-direction is given by equation (5) with equation (3). Thus, the equations of motion (divided by a constant *α*) are given by

9 $$\{\begin{array}{c}0=k\frac{{{\rm{\partial }}}^{2}}{{\rm{\partial }}{x}^{2}}u(x,t)+c-l{E}_{0}u(x,t)-l\eta \frac{{\rm{\partial }}}{{\rm{\partial }}t}u(x,t)\\ 0=k\frac{{{\rm{\partial }}}^{2}}{{\rm{\partial }}{x}^{2}}u(x,t)+c-L{E}_{0}u(x,t)-L\eta \frac{{\rm{\partial }}}{{\rm{\partial }}t}u(x,t),\end{array}$$

for the rear and front sides, respectively. Here, *k* ≡ *l* ^2^ *K*/*α* and *c* are independent of position (*x*) and time (*t*). To seek a solution corresponding to a constant-velocity crack propagation, we substitute a solution of the form *u*(*x*, *t*) = *f*(*x* − *Vt*) into equations (9) to obtain linear ordinary differential equations (ODE):

10 $$\{\begin{array}{c}0=c-l{E}_{0}\,f(x)+lV\eta \,f^{\prime} (x)+kf^{\prime\prime} (x)\\ 0=c-L{E}_{0}\,f(x)+LV\eta \,f^{\prime} (x)+kf^{\prime\prime} (x),\end{array}$$

for the rear and front sides, respectively.

We can solve equation (10) with appropriate boundary conditions at *x* = ±∞ and matching conditions for the rear and front solutions at the crack tip (See Supplementary Section II for the details). As a result, we find that crack propagates only in the range $1/\sqrt{N} < \tilde{\varepsilon } < 1$ or equivalently *w*_0_ < *w* < *w*_0_ *N*, and the velocity is exactly given by

11 $$\frac{V}{{V}_{0}}=\frac{N{\tilde{\varepsilon }}^{2}-1}{\sqrt{N\tilde{\varepsilon }(1-\tilde{\varepsilon })(N\tilde{\varepsilon }-1)}},$$

with $\tilde{\varepsilon }\equiv \varepsilon /{\varepsilon }_{c}=\sqrt{w/({w}_{0}N)}$. Equation (11) for the model consisting of Kelvin-Voigt elements is the counterpart of equation (6) for the model consisting of Zener elements. In fact, by taking the limit *λ* → ∞ in equation (6), we have equation (11), which does not reproduce the velocity jump (Fig. 4e), unlike equation (6).

We next briefly describe how to generalize the above procedure to the model consisting of Zener elements illustrated in Fig. 2e. The counterparts of equation (9) is expressed as the following set of equation of motion, in which two variables *u* and *u*_2_ are coupled:

12 $$\{\begin{array}{c}0=k\frac{{{\rm{\partial }}}^{2}}{{\rm{\partial }}{x}^{2}}u(x,t)+c-l{E}_{0}u(x,t)-l\eta \frac{{\rm{\partial }}}{{\rm{\partial }}t}{u}_{2}(x,t)\\ 0=k\frac{{{\rm{\partial }}}^{2}}{{\rm{\partial }}{x}^{2}}u(x,t)+c-L{E}_{0}u(x,t)-L\eta \frac{{\rm{\partial }}}{{\rm{\partial }}t}{u}_{2}(x,t).\end{array}$$

Here, ${E}_{1}{u}_{1}=\eta \frac{\partial }{\partial t}{u}_{2}$, with the elongation of dashpot *u*_2_ and the total elongation *u* = *u*_1_ + *u*_2_. By noting the relation $u=\frac{\eta }{{E}_{1}}\frac{\partial }{\partial t}{u}_{2}+{u}_{2}$, the set of equation of motion can be written only in terms of *u*_2_ by removing the variables *u* and *u*_1_. Substituting *u*_2_(*x*, *t*) = *f*_2_(*x* − *Vt*) into equation (12) as before, we obtain a third-order linear ODE for *f*_2_, which can be solved under the boundary conditions including matching conditions for the rear and front solutions. As a result, we obtain equation (6) together with equation (7), which is a characteristic equation for the third-order linear ODE for *f*_2_. We explain the details of the derivation in Supplementary Section III.

### Theorems

We give the theorems used to obtain the main result in equation (6) and to plot Fig. 3. The details and proofs of the theorems are relegated to Supplementary Section III.

Through the procedures explained above, we obtain the following third-order linear ODE and boundary conditions, which describe constant-velocity crack propagation:

13 $$\{\begin{array}{cc}0=f(\chi )-\frac{\nu \lambda }{\lambda -1}f^{\prime} (\chi )-Nf^{\prime\prime} (\chi )+\frac{\nu N}{\lambda -1}f{\rm{^{\prime} }}{\rm{^{\prime} }}{\rm{^{\prime} }}(\chi ) & {\rm{f}}{\rm{o}}{\rm{r}}\,\chi  < 0\quad (\text{rear}\,\text{side})\\ 0=f(\chi )-\varepsilon -\frac{\nu \lambda }{\lambda -1}f^{\prime} (\chi )-f^{\prime\prime} (\chi )+\frac{\nu }{\lambda -1}f{\rm{^{\prime} }}{\rm{^{\prime} }}{\rm{^{\prime} }}(\chi ) & {\rm{f}}{\rm{o}}{\rm{r}}\,0\le \chi \quad (\text{front}\,\text{side}),\end{array}$$

14 $$\{\begin{array}{c}f(-\mathrm{0)}-\frac{\nu }{\lambda -1}f^{\prime} (-\mathrm{0)}=f(+\mathrm{0)}-\frac{\nu }{\lambda -1}f^{\prime} (+0)=\frac{N\varepsilon -{\varepsilon }_{c}}{N-1};\\ f^{\prime} (-\mathrm{0)}={f}^{\text{'}}(+\mathrm{0);}\quad \quad \quad \quad \quad \quad \quad \quad \quad \quad \quad \quad \quad f^{\prime\prime} (-\mathrm{0)}=f^{\prime\prime} (+\mathrm{0);}\\ f(-\infty )=\mathrm{0;}\quad \quad \quad \quad \quad \quad \quad \quad \quad \quad \quad \quad \quad \quad \quad \,\,f(+\infty )=\varepsilon .\end{array}$$

Here, we introduce the dimensionless parameters *ν* ≡ *V*/*V*_0_ and *χ* ≡ *x*/*x*_0_. The latter is the distance along the *x*-axis from the crack tip normalized by the reference length scale ${x}_{0}\equiv l\sqrt{(1-\frac{1}{N})\frac{\mu }{2{E}_{0}}}$.

The relation between initially applied strain and crack-propagation velocity is given by the following theorem:

*Theorem 1*. *If* *equations* (13) *and* (14) *hold*, *then*

15 $$\tilde{\varepsilon }\equiv \frac{\varepsilon }{{\varepsilon }_{c}}=\frac{\frac{\nu }{\lambda -1}(\frac{N}{{\xi }_{1}{\xi }_{N}}+\lambda )+{\xi }_{1}+{\xi }_{N}}{\frac{\nu }{\lambda -1}(N+\frac{N}{{\xi }_{1}{\xi }_{N}}+\lambda -1)+N{\xi }_{1}+{\xi }_{N}}.$$

Asymptotic forms in low- and high-velocity regimes are given by the following theorem:

*Theorem 2*. *If λ* > *1 and N* > 1, *then*

16 $$\tilde{\varepsilon }(\nu )=\frac{1}{\sqrt{N}}+\frac{\sqrt{N}-1}{2N}\nu +O({\nu }^{2})\quad (\nu \to \mathrm{0),}$$

*and*

17 $$\tilde{\varepsilon }(\nu )=\frac{\lambda }{\sqrt{N}+\lambda -1}-\frac{{(\lambda -1)}^{2}(\sqrt{N}-1)\,(\sqrt{N}+2)}{2\nu \sqrt{\lambda }{(\sqrt{N}+\lambda -1)}^{2}}+O(\frac{1}{{\nu }^{2}})\quad (\nu \to \infty \mathrm{)}.$$

The existence condition of the velocity jump is given by the following theorem:

*Theorem 3*. *If* 1 < *λ* < ∞, 1 < *N* < ∞, *and* $\frac{\lambda -1}{\sqrt{\lambda }}{V}_{0} < V < \sqrt{N}{V}_{0}$, *then the initially applied strain ε* = *ε*(*ν*, *λ*, *N*) *is bounded as follows*:

18 $$\sqrt{\frac{\lambda }{N}}(1-\frac{\lambda -1}{\nu \sqrt{\lambda }}-\frac{\nu \sqrt{\lambda }}{N}) < \tilde{\varepsilon }\equiv \frac{\varepsilon }{{\varepsilon }_{c}} < \sqrt{\frac{\lambda }{N}}(1+\frac{\nu }{\sqrt{N}}).$$

According to Theorem 3, we have the approximate expression $\tilde{\varepsilon }\simeq \sqrt{\frac{\lambda }{N}}$ in the range of *ν*,

19 $$\frac{\lambda -1}{\sqrt{\lambda }}\ll \nu \ll \sqrt{N}.$$

### Ψ_soft_ and Ψ_hard_ for short and long Zener elements

We give explicit forms of the parameters Ψ_soft_ and Ψ_hard_ used to plot Fig. 5b,c. By using results obtained in Supplementary Section III, we have Ψ_soft_ and Ψ_hard_ for “short” Zener elements as

20 $$\{\begin{array}{c}{{\rm{\Psi }}}_{{\rm{s}}{\rm{o}}{\rm{f}}{\rm{t}}}^{{\rm{s}}{\rm{h}}{\rm{o}}{\rm{r}}{\rm{t}}}=\frac{\lambda -1}{\lambda }\frac{{\xi }_{1}}{\nu }[\frac{\mathop{\varepsilon }\limits^{ \sim }}{1-\mathop{\varepsilon }\limits^{ \sim }}{e}^{\chi /{\xi }_{1}}+1]\\ {{\rm{\Psi }}}_{{\rm{h}}{\rm{a}}{\rm{r}}{\rm{d}}}^{{\rm{s}}{\rm{h}}{\rm{o}}{\rm{r}}{\rm{t}}}=\frac{\nu }{(\lambda -1){\xi }_{1}}{[\frac{\mathop{\varepsilon }\limits^{ \sim }}{1-\mathop{\varepsilon }\limits^{ \sim }}\cdot \frac{(\lambda -1){\xi }_{1}+\nu }{(\lambda -1){\xi }_{1}+\lambda \nu }{e}^{\chi /{\xi }_{1}}+1]}^{-1},\end{array}$$

respectively. Equations (20) together with equation (6) give contour plots in Fig. 5b.

Expressions for the “long” Zener elements are different depending on whether the element is located at the front or rear side of the crack tip. On the front side, we have

21 $$\{\begin{array}{c}{{\rm{\Psi }}}_{{\rm{s}}{\rm{o}}{\rm{f}}{\rm{t}}}^{{\rm{l}}{\rm{o}}{\rm{n}}{\rm{g}},{\rm{f}}{\rm{r}}{\rm{o}}{\rm{n}}{\rm{t}}}=\frac{\lambda -1}{\lambda }\frac{{\xi }_{1}}{\nu }[\frac{(N-1)\mathop{\varepsilon }\limits^{ \sim }}{1-\mathop{\varepsilon }\limits^{ \sim }}{e}^{\chi /{\xi }_{1}}-1]\\ {{\rm{\Psi }}}_{{\rm{h}}{\rm{a}}{\rm{r}}{\rm{d}}}^{{\rm{l}}{\rm{o}}{\rm{n}}{\rm{g}},{\rm{f}}{\rm{r}}{\rm{o}}{\rm{n}}{\rm{t}}}=\frac{\nu }{(\lambda -1){\xi }_{1}}{[\frac{(N-1)\mathop{\varepsilon }\limits^{ \sim }}{1-\mathop{\varepsilon }\limits^{ \sim }}\cdot \frac{(\lambda -1){\xi }_{1}+\nu }{(\lambda -1){\xi }_{1}+\lambda \nu }{e}^{\chi /{\xi }_{1}}-1]}^{-1}.\end{array}$$

On the rear side, we have

22 $$\{\begin{array}{c}{{\rm{\Psi }}}_{{\rm{s}}{\rm{o}}{\rm{f}}{\rm{t}}}^{{\rm{l}}{\rm{o}}{\rm{n}}{\rm{g}},{\rm{r}}{\rm{e}}{\rm{a}}{\rm{r}}}\equiv \frac{\lambda -1}{\lambda }\frac{{\mathscr{E}}}{|\dot{{\mathscr{E}}}|}\\ {{\rm{\Psi }}}_{{\rm{h}}{\rm{a}}{\rm{r}}{\rm{d}}}^{{\rm{l}}{\rm{o}}{\rm{n}}{\rm{g}},{\rm{r}}{\rm{e}}{\rm{a}}{\rm{r}}}\equiv \frac{1}{\lambda -1}\frac{|\dot{\sigma }|}{\sigma },\end{array}$$

where

23 $$\{\begin{array}{c}{\mathscr{E}}={C}_{0}{\sum }_{i=1}^{2}{D}_{i}[1+\frac{\nu }{(\lambda -1){\xi }_{N,i}}]{e}^{-\chi /{\xi }_{N,i}}\\ \dot{{\mathscr{E}}}={C}_{0}{\sum }_{i=1}^{2}{D}_{i}[1+\frac{\nu }{(\lambda -1){\xi }_{N,i}}]\frac{\nu }{{\xi }_{N,i}}{e}^{-\chi /{\xi }_{N,i}}\\ \sigma ={C}_{0}{\sum }_{i=1}^{2}{D}_{i}[1+\frac{\lambda \nu }{(\lambda -1){\xi }_{N,i}}]{e}^{-\chi /{\xi }_{N,i}}\\ \dot{\sigma }={C}_{0}{\sum }_{i=1}^{2}{D}_{i}[1+\frac{\lambda \nu }{(\lambda -1){\xi }_{N,i}}]\frac{\nu }{{\xi }_{N,i}}{e}^{-\chi /{\xi }_{N,i}}.\end{array}$$

Note that *χ* < 0 on the rear side. Here, *ξ*_*N*,1_, *ξ*_*N*,2_, and *ξ*_*N*_ with *ξ*_*N*,1_ < *ξ*_*N*,2_ < 0 < *ξ*_*N*_ are the solutions of the cubic equation (7) for *ξ* with ${D}_{1}=\frac{{\gamma }_{2}+1}{{\gamma }_{1}({\gamma }_{2}-{\gamma }_{1})}$ and ${D}_{2}=-\frac{{\gamma }_{1}+1}{{\gamma }_{2}({\gamma }_{2}-{\gamma }_{1})}$ where *γ*_1_ ≡ −*ξ*_1_/*ξ*_*N*,1_ and *γ*_2_ ≡ −*ξ*_1_/*ξ*_*N*,2_. ${C}_{0}\equiv \frac{{\varepsilon }_{c}-\varepsilon }{N-1}\cdot \frac{(\lambda -\mathrm{1)}{\xi }_{1}}{(\lambda -\mathrm{1)}{\xi }_{1}+\nu }$ is a positive constant. Equations (21) and (22) together with equation (6) give contour plots in Fig. 5c.

## Electronic supplementary material

Supplementary Information
