# Supplementary material for: Exactly solvable model for a velocity jump observed in crack propagation in viscoelastic solids
Source: Sci Rep. 2017 Aug 14;7:8065. doi: 10.1038/s41598-017-07214-8 (PMC5556105; doi:10.1038/s41598-017-07214-8)
Supplement: Supplementary file 1 — Supplementary Information [file 41598_2017_7214_MOESM1_ESM.pdf]

Supplementary Information for:
Exactly solvable model for a velocity jump observed in crack propagation in viscoelastic solids

Naoyuki Sakumichi[1] and Ko Okumura[1,2]

[1] *Soft Matter Center, Ochanomizu University, Bunkyo-ku, Tokyo 112-8610, Japan*
[2] *Department of Physics, Ochanomizu University, Bunkyo-ku, Tokyo 112-8610, Japan*

(Dated: August 2, 2017)

In this note, we explain the details of the formulation of the model and the derivation of the analytical solutions given in the main text. This note is organized as follows. In Sec. I, we describe a lattice model for the fixed-grip crack propagation in viscoelastic sheets with incorporating Kelvin-Voigt elements for the interaction between the lattice points. In Sec. II, we construct a model for which an analytical solution is available for a constant-velocity crack propagation, by simplifying the ordinary lattice model introduced in Sec. I. For the model consisting of Kelvin-Voigt elements, we derive an analytical expression for the crack-propagation velocity as a function of the initially applied energy density and show that the model does not exhibit the velocity jump. In Sec. III, we replace the Kelvin-Voigt interaction with the Zener interaction in the model. For the model consisting of Zener elements, we derive an analytical expression for the crack-propagation velocity as a function of the initially applied energy density and reveal that the model exhibits the velocity jump. In Sec. IV, from the analytical solution, we derive the existence condition of the velocity jump, together with simple relations useful for controlling crack propagation in developing tough materials.

Contents

I. Lattice model consisting of Kelvin-Voigt elements 1

II. Minimal model consisting of Kelvin-Voigt elements 3
A. Construction of a minimal model 3
B. Derivation of an exact solution of crack propagation with a constant velocity 5

III. Minimal model consisting of Zener elements 1: the basic equations and exact solution 7
A. Generalization of viscoelastic interaction: equations of motion in the y-direction 7
B. Derivation of the exact solution of crack propagation with a constant velocity 8

IV. Minimal model consisting of Zener elements 2: low- and high-velocity regimes and velocity jump 11
A. Low- and high-velocity regimes 11
B. Existence condition of the velocity jump 13

References 17

I. LATTICE MODEL CONSISTING OF KELVIN-VOIGT ELEMENTS

We construct a lattice model for crack propagation in a viscoelastic sheet, which satisfies the following conditions:

- The viscoelastic sheet is always on the x-y plane. Under zero strain, the height (in the y-direction) and thickness (in the z-direction) of the sheet are L and h, respectively, whereas the width (in the x-direction) is much larger than L.
- The fixed strain ε $(\equiv \Delta L/L \geq 0)$ is applied in the y-direction.
- A line crack propagates in the positive x-direction.

We consider a two-dimensional model on an $N_1 \times N_2$ rectangular lattice where N_1 is (countably) infinite and N_2 is even. The lattice points are labeled by the index $\mathbf{n} = (n_1, n_2)$ where $n_1 \in \mathbb{Z} \equiv \{\dots, -1, 0, 1, \dots\}$ and $n_2 \in \{1, 2, \dots, N_2\}$. At $\varepsilon = 0$, the lattice constant in the x- and y-direction are a and b, respectively. In other words, under zero strain, the height and width of the sheet are $aN_1 (= \infty)$ and $bN_2 (= L)$, respectively. The position of the lattice point labeled by the index $\mathbf{n}$ is given by $\mathbf{r}_0(\mathbf{n}) = (an_1, bn_2 - L/2)$. When the top and bottom edges of

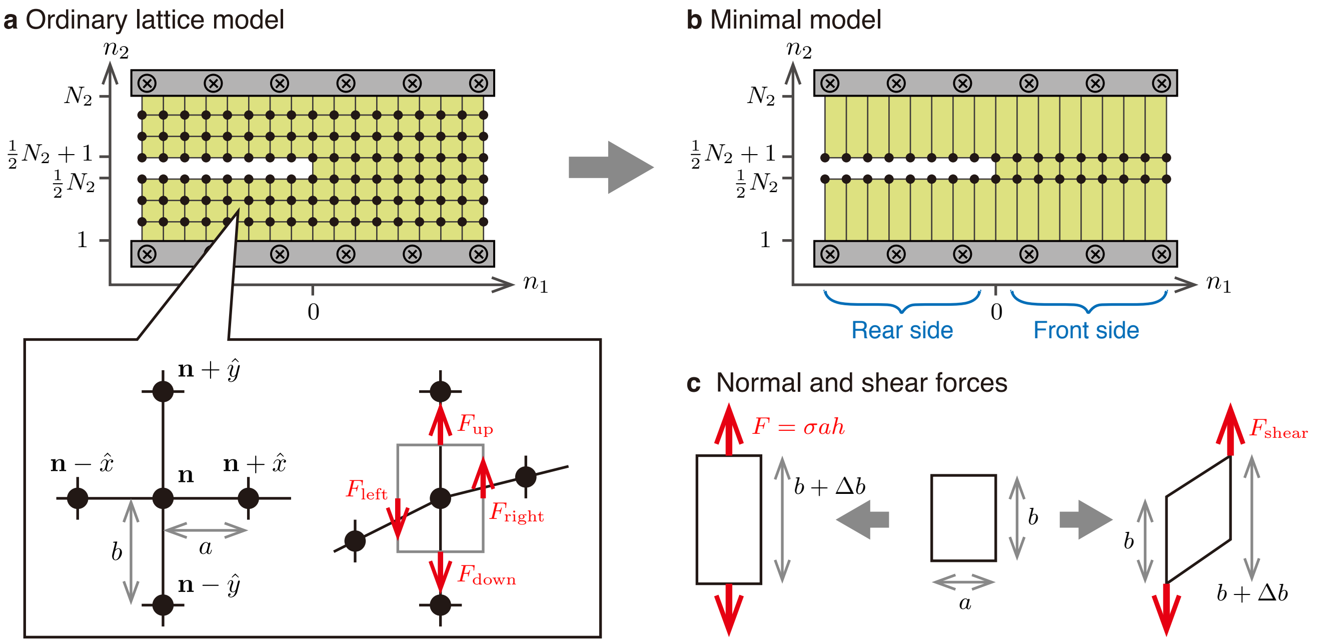

FIG. S1: **Ordinary and minimal lattice models for the fixed-grip crack propagation.** **a**, Lattice model with a rectangular unit cell under zero strain. The lattice point is specified by two integers $\mathbf{n} = (n_1, n_2)$. The top and bottom boundaries are located at $n_2 = 1$ and $n_2 = N_2$, respectively, and the two surfaces of the line crack are located at $n_2 = N_2/2$ and $n_2 = N_2/2 + 1$. In the box, magnified views on a lattice point and its nearest neighbor points with and without deformation are shown together with the indices of the points, the lattice constants a and b, and associated forces. **b**, Coarse-grained semi-lattice model obtained by removing lattice points except for the ones located at $n_2 = N_2/2$ and $n_2 = N_2/2 + 1$. **c**, Elementary deformation modes of a unit cell in the rectangular lattice with lattice spacings a and b.

the sheet are clamped and stretched in the y-direction ($\varepsilon > 0$), we set the coordinate of the points on the edges to $\mathbf{r}_\varepsilon(n_1, 1) = (an_1, -L(1+\varepsilon)/2)$ and $\mathbf{r}_\varepsilon(n_1, N_2) = (an_1, L(1+\varepsilon)/2)$. The displacement of the lattice point $\mathbf{n}$ is given by $\mathbf{r}_\varepsilon(\mathbf{n}) - \mathbf{r}_0(\mathbf{n}) \equiv \mathbf{u}(\mathbf{n}) = (u_1(\mathbf{n}), u_2(\mathbf{n}))$. Note that, in the main text, we use u_i to denote the y-coordinate.

In the model, every lattice point interacts with the nearest neighbor points (at most four points) by a minimal coupling. The tensile and shear stresses are given as $F_{\text{tensile}}/(ah) = E\Delta b/b$ and $F_{\text{shear}}/(bh) = G\Delta b/a$, respectively, for the deformation characterized by Δb (See, Fig. S1 for the case in which the unit cell deforms in the y-direction). Here, we have introduced Young's modulus E and the shear modulus G. The elastic energy of the sheet with the minimal coupling is then given by

$$U[u_\mu(\mathbf{n})] = h \sum_{n_1=-\infty}^{\infty} \sum_{n_2=1}^{N_2} \sum_{\mu=1}^{2} \sum_{\nu=1}^{2} \frac{1}{2} C_{\mu\nu} \left[u_\mu(\mathbf{n}+\hat{\nu}) - u_\mu(\mathbf{n})\right]^2, \tag{I.1}$$

where $C_{11} = C_{22} = Ea/b$ and $C_{12} = C_{21} = Gb/a$. Here, $\hat{1} \equiv \hat{x} \equiv (1,0)$ and $\hat{2} \equiv \hat{y} \equiv (0,1)$, $\delta_{\mu\nu}$ is the Kronecker delta, and the summation extends over all the bonds on the sheet. We note that Poisson's ratio of this system is zero, i.e., $G = E/2$ because Eq. (I.1) does not contain the coupling between the displacements in the x- and y-directions. Moreover, assuming that the displacements in the x-direction $u_1(\mathbf{n})$ for arbitrary $\mathbf{n}$ are zero at the initial time, forces in the x-direction are always zero, and thus, every lattice point does not move in the x-direction. Then, in the following, we consider the motions and forces only in the y-direction.

To construct the equation of motion for the system, we consider tensile and shear forces, together with viscous forces. As illustrated in Fig. S1, the tensile and shear forces acting on the lattice point $\mathbf{n}$ in the y-direction are given, respectively, as follows:

$$F_{\text{up}} - F_{\text{down}} = Eah\left[\frac{u_2(\mathbf{n}+\hat{y}) - u_2(\mathbf{n})}{b} - \frac{u_2(\mathbf{n}) - u_2(\mathbf{n}-\hat{y})}{b}\right] = Eabh\frac{\Delta^2}{\Delta y^2}u_2(\mathbf{n}), \tag{I.2}$$

$$F_{\text{right}} - F_{\text{left}} = Gbh\left[\frac{u_2(\mathbf{n}+\hat{x}) - u_2(\mathbf{n})}{a} - \frac{u_2(\mathbf{n}) - u_2(\mathbf{n}-\hat{x})}{a}\right] = Gabh\frac{\Delta^2}{\Delta x^2}u_2(\mathbf{n}). \tag{I.3}$$

Here, we have introduced the notations, $\frac{\Delta^2}{\Delta x^2} f(\mathbf{n}) \equiv [f(\mathbf{n}+\hat{x}) - 2f(\mathbf{n}) + f(\mathbf{n}-\hat{x})]/a^2$ and $\frac{\Delta^2}{\Delta y^2} f(\mathbf{n}) \equiv [f(\mathbf{n}+\hat{y}) - 2f(\mathbf{n}) + f(\mathbf{n}-\hat{y})]/b^2$, which correspond to the second-order partial derivatives in the continuum limits, $a \to 0$ and $b \to 0$, respectively. When a Kelvin-Voigt element is employed for the interaction in the y-direction, the following viscous term should be added:

$$F_{\mathrm{up}}^{(\eta)} - F_{\mathrm{down}}^{(\eta)} = \eta a h \left[\frac{\frac{\partial}{\partial t} u_2(\mathbf{n}+\hat{y}) - \frac{\partial}{\partial t} u_2(\mathbf{n})}{b} - \frac{\frac{\partial}{\partial t} u_2(\mathbf{n}) - \frac{\partial}{\partial t} u_2(\mathbf{n}-\hat{y})}{b} \right] = \eta a b h \frac{\Delta^2}{\Delta y^2} \frac{\partial}{\partial t} u_2(\mathbf{n}). \tag{I.4}$$

In this way, the equation of motion of the lattice point $\mathbf{n}$ in the y-direction is given by

$$\begin{aligned} m \frac{\partial^2}{\partial t^2} u_2(\mathbf{n}) &= F_{\mathrm{right}} - F_{\mathrm{left}} + F_{\mathrm{up}} - F_{\mathrm{down}} + F_{\mathrm{up}}^{(\eta)} - F_{\mathrm{down}}^{(\eta)} \\ &= abh \left[G \frac{\Delta^2}{\Delta x^2} u_2(\mathbf{n}) + E \frac{\Delta^2}{\Delta y^2} u_2(\mathbf{n}) + \eta \frac{\Delta^2}{\Delta y^2} \frac{\partial}{\partial t} u_2(\mathbf{n}) \right]. \end{aligned} \tag{I.5}$$

Assuming that the inertial term $m \frac{\partial^2}{\partial t^2} u_2(\mathbf{n})$ is negligible (the overdamped limit), we obtain the equation of motion per unit volume in the following form:

$$0 = G \frac{\Delta^2}{\Delta x^2} u_2(\mathbf{n}) + E \frac{\Delta^2}{\Delta y^2} u_2(\mathbf{n}) + \eta \frac{\Delta^2}{\Delta y^2} \frac{\partial}{\partial t} u_2(\mathbf{n}). \tag{I.6}$$

II. MINIMAL MODEL CONSISTING OF KELVIN-VOIGT ELEMENTS

A. Construction of a minimal model

In this section, we construct a minimal model for crack propagation in viscoelastic sheets as shown in Fig. S1b, by ignoring all the lattice points except for the lattice points at $n_2 = N_2/2$ and $n_2 = N_2/2+1$. The original set of variables $\{u_2(\mathbf{n})\}$ is now represented by a much smaller set, $\{u_2(i, N_2/2)\} \cup \{u_2(i, N_2/2+1)\}$ for $i \in \mathbb{Z} \equiv \{\dots, -1, 0, 1, \dots\}$. For simplicity, we assume that the sheet is always symmetric about the x axis, and thus the lattice points on the upper side, $u_i \equiv u_2(i, N_2+1)$ for $i \in \mathbb{Z}$, completely describe the dynamics of the present model.

In the following discussion, it is important to distinguish three types of strain in the y-direction (see Fig. S2a; here and hereafter, we set $l \equiv b$ and use l instead of b):

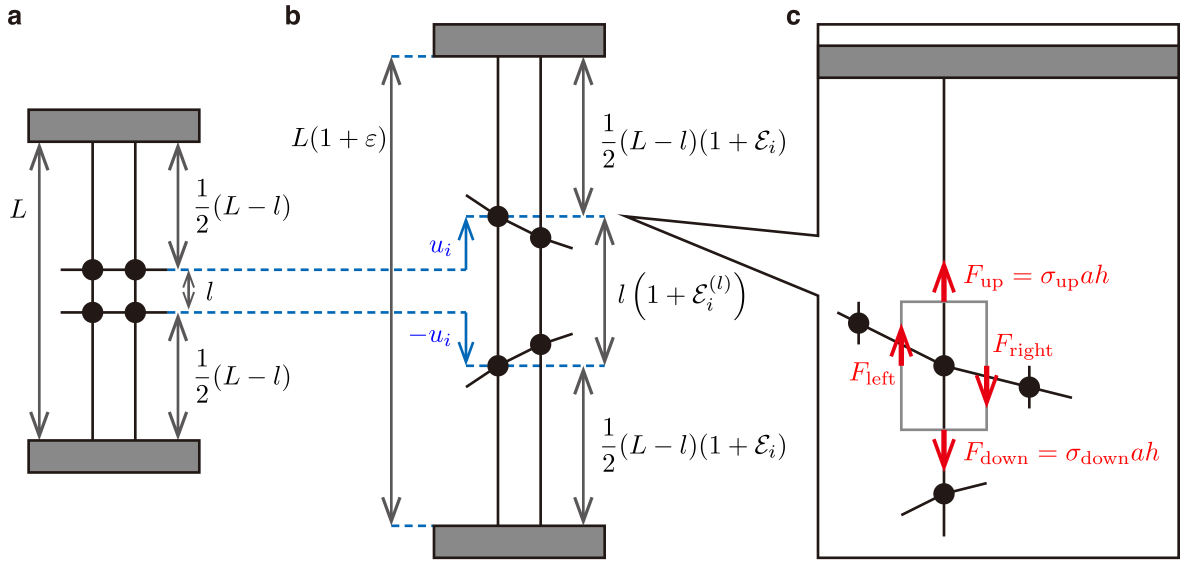

FIG. S2: **Three types of strain (ε, $\mathcal{E}_i(t)$, and $\mathcal{E}_i^{(l)}(t)$) and forces acting on a lattice point. a,** The proposed minimal lattice model under zero strain. We call the springs of length $(L-l)/2$ and those of length l the long and short springs, respectively. **b,** The model under the finite strain ε. **c,** Forces acting on a lattice point in the model.

(i) ε: the initially applied strain;

(ii) $\mathcal{E}_i(t)$: the strain of the i-th upper (or lower) "long spring," i.e., the i-th spring of natural length $(L-l)/2$ directly connected to the top (or bottom) boundary (the strains of the upper and lower long springs are the same so that in the following we consider only the upper ones and "long springs" indicate the upper ones);

(iii) $\mathcal{E}_i^{(l)}(t) \equiv [u_2(i, N_2+1) - u_2(i, N_2)]/l = 2u_i/l$: the strain of the i-th "short spring," i.e., the i-th spring of natural length l located at the center in the y-direction (provided that the i-th short spring exists).

Here, ε is a constant, but $\mathcal{E}_i(t)$ and $\mathcal{E}_i^{(l)}(t)$ depend on time t. We mainly use $\mathcal{E}_i(t)$ to describe the dynamics of crack propagation because $\mathcal{E}_i^{(l)}(t)$ is expressed by $\mathcal{E}_i(t)$ by the following relation (see Fig. S2b):

$$L\varepsilon = (L-l)\mathcal{E}_i(t) + l\mathcal{E}_i^{(l)}(t). \tag{II.1}$$

Note that $\mathcal{E}_i(t)$ is much smaller than $\mathcal{E}_i^{(l)}(t)$ provided that the i-th short spring exists because "the spring constant" of long springs is much smaller than that of the short springs (the corresponding Young's moduli are the same for the short and long springs). To realize crack propagation, we remove the i-th short spring when $\mathcal{E}_i^{(l)}(t) \geq \varepsilon_c$. At places far from the crack tip, $\mathcal{E}_i(t)$ approaches a constant value and the value depends on whether the i-th long spring is located on the front (i.e., right) or rear (i.e., left) side of the tip of a crack propagating in the positive x-direction (see Fig. S1b):

$$\begin{cases} \lim\limits_{i\to-\infty} \mathcal{E}_i(t) = 0 & \text{rear side} \\ \lim\limits_{i\to+\infty} \mathcal{E}_i(t) = \lim\limits_{i\to+\infty} \mathcal{E}_i^{(l)}(t) = \varepsilon. & \text{front side} \end{cases} \tag{II.2}$$

We construct the equation of motion in the y-direction for the i-th lattice point characterized by $u_i(t)$ in the minimal model. First, we consider tensile stresses on the basis of Eq. (I.6); as illustrated in Fig. S2c, considering the elongational deformation in the y-direction of the i-th long and short springs, we obtain the tensile stresses acting on the i-th lattice point in the following form:

$$\begin{cases} \dfrac{F_{\mathrm{up},i}(t)}{ah} = E_0\mathcal{E}_i(t) \\ \dfrac{F_{\mathrm{down},i}(t)}{ah} = E_0\mathcal{E}_i^{(l)}(t), \end{cases} \tag{II.3}$$

from which, we have $F_{\mathrm{up},i}(t) - F_{\mathrm{down},i}(t) = E_0\left(\mathcal{E}_i(t) - \varepsilon\right) ahL/l$ by virtue of Eq. (II.1). Here, we explicitly show the subscript 0 for the Young's modulus, E_0, whereas we have omitted the subscript in Sec. I, i.e., $E \equiv E_0$. This is because we should distinguish the two springs E_0 and E_1 in a Zener element, which is a generalization of a Kelvin-Voigt element and will be considered in Secs. III and IV. Second, we consider shear stresses; as illustrated in Fig. S2c, considering the shear deformation in the y-direction, we obtain $F_{\mathrm{right},i} - F_{\mathrm{left},i} = alh\mu\frac{\Delta^2}{\Delta x^2}u_i(t)$. Note that shear modulus G for the ordinary lattice model (Fig. S1a, discussed in Sec. I) is replaced by the "effective" shear modulus μ for the minimal model (Fig. S1b). This is because the shear modulus for the minimal model is considered to effectively represent all the forces acting on the decimated points from the nearest neighbor points located in the x-directions, with a spirit similar to the one employed in renormalization [S1] in statistical physics. Third, combining the tensile and shear stresses together with the viscous terms, we obtain the equations of motion in the y-direction for the i-th lattice point of mass m, by noting that $F_{\mathrm{down},i}(t)$ and $F^{(\eta)}_{\mathrm{down},i}(t)$ are missing on the rear side of the crack tip:

$$\begin{cases} m\dfrac{\partial^2}{\partial t^2}u_i(t) = F_{\mathrm{up},i} + F^{(\eta)}_{\mathrm{up},i} + F_{\mathrm{right},i} - F_{\mathrm{left},i} = ah\left[E_0\mathcal{E}_i(t) + \eta\dfrac{\partial}{\partial t}\mathcal{E}_i(t)\right] + alh\mu\dfrac{\Delta^2}{\Delta x^2}u_i(t) & \text{rear side} \\ m\dfrac{\partial^2}{\partial t^2}u_i(t) = F_{\mathrm{up},i} + F^{(\eta)}_{\mathrm{up},i} - F_{\mathrm{down},i} - F^{(\eta)}_{\mathrm{down},i} + F_{\mathrm{right},i} - F_{\mathrm{left},i} & \\ \qquad = ah\dfrac{L}{l}\left[E_0\left(\mathcal{E}_i(t) - \varepsilon\right) + \eta\dfrac{\partial}{\partial t}\mathcal{E}_i(t)\right] + alh\mu\dfrac{\Delta^2}{\Delta x^2}u_i(t) & \text{front side.} \end{cases} \tag{II.4}$$

Since Eq. (II.1) can be expressed as $\mathcal{E}_i(t) = [L\varepsilon - 2u_i(t)]/(L-l)$, we can rewrite the above equations of motion in terms of $\mathcal{E}_i(t)$ by removing the dynamic variable $u_i(t)$. Finally, assuming that the inertial term is negligible (the overdamped limit), we obtain the equations of motion for the field $\mathcal{E}_i(t)$:

$$\begin{cases} 0 = E_0\mathcal{E}_i(t) + \eta\dfrac{\partial}{\partial t}\mathcal{E}_i(t) - \dfrac{1}{2}l(L-l)\mu\dfrac{\Delta^2}{\Delta x^2}\mathcal{E}_i(t) & \text{rear side} \\ 0 = \dfrac{L}{l}E_0\left(\mathcal{E}_i(t) - \varepsilon\right) + \dfrac{L}{l}\eta\dfrac{\partial}{\partial t}\mathcal{E}_i(t) - \dfrac{1}{2}l(L-l)\mu\dfrac{\Delta^2}{\Delta x^2}\mathcal{E}_i(t) & \text{front side.} \end{cases} \tag{II.5}$$

We here make three comments on the equations of motion (II.5). (i) When we neglect shear force ($\mu = 0$), the two expressions on the front and rear sides reduce to the same equation of motion (except for the equilibrium position), which describes the Kelvin-Voigt dynamics. (ii) The "second-derivative" term $E_0 \frac{\Delta^2}{\Delta y^2} u_2(\mathbf{n})$ in Eq. (I.6) has been replaced by the strain-field term, $E_0 \mathcal{E}_i(t)$ (rear side) or $\frac{L}{l} E_0 \left(\mathcal{E}_i(t) - \varepsilon\right)$ (front side) in Eq. (II.5) with proportional constants. (iii) The net strains of long springs on the rear and front sides are different and given by $\mathcal{E}_i(t)$ and $\mathcal{E}_i(t) - \varepsilon$, respectively, because $\lim_{i \to -\infty} \mathcal{E}_i(t) = 0$ and $\lim_{i \to -\infty} \mathcal{E}_i(t) = \varepsilon$ on the rear and front sides, respectively (see Eq. (II.2)).

To derive the equations to be solved analytically, we take the continuum limit of Eq. (II.5) in the x-direction, $a \to 0$. In this limit, the finite difference $\frac{\Delta^2}{\Delta x^2}$ is replaced by the derivative $\frac{\partial^2}{\partial x^2}$ and the discrete strain field $\mathcal{E}_i(t)$ by the continuum strain field $\mathcal{E} \equiv \mathcal{E}(\tau, \chi)$ where we have introduced the dimensionless parameters, $\tau = t/t_0$, $\chi = x/x_0$ with

$$t_0 \equiv \frac{\eta}{E_0} \quad \text{and} \quad x_0 \equiv l \sqrt{\left(1 - \frac{l}{L}\right) \frac{\mu}{2E_0}}. \tag{II.6}$$

In this way, the above discrete version of the equations motion (II.5) is replaced by the following continuum equations:

$$\begin{cases} 0 = \mathcal{E} + \dot{\mathcal{E}} - N\mathcal{E}'' & \text{rear side} \\ 0 = (\mathcal{E} - \varepsilon) + \dot{\mathcal{E}} - \mathcal{E}''. & \text{front side} \end{cases} \tag{II.7}$$

Here, we have introduced the notations, $\dot{\mathcal{E}} \equiv \frac{\partial}{\partial \tau} \mathcal{E}(\tau, \chi)$, $\mathcal{E}'' \equiv \frac{\partial^2}{\partial \chi^2} \mathcal{E}(\tau, \chi)$, and

$$N \equiv \frac{L}{l}. \tag{II.8}$$

B. Derivation of an exact solution of crack propagation with a constant velocity

In this subsection, we solve Eq. (II.7) for the static case ($V = 0$) and for the dynamic case in which a crack propagates with a constant velocity ($0 < V < \infty$), by seeking a solution of the form $\mathcal{E}(\tau, \chi) = f(\chi - \nu\tau)$. Here, we have introduced the dimensionless velocity of crack propagation,

$$\nu = \frac{V}{V_0}, \tag{II.9}$$

with V_0 defined by

$$V_0 \equiv \frac{x_0}{t_0} = \frac{l}{\eta} \sqrt{\left(1 - \frac{l}{L}\right) \frac{E_0 \mu}{2}}. \tag{II.10}$$

Substituting $f(\chi - \nu\tau)$ into Eq. (II.7), we have second-order linear ordinary differential equations:

$$\begin{cases} 0 = f(\chi) - \nu f'(\chi) - N f''(\chi) & \text{for } \chi < 0 \quad \text{(rear side)} \\ 0 = f(\chi) - \varepsilon - \nu f'(\chi) - f''(\chi) & \text{for } 0 \le \chi \quad \text{(front side).} \end{cases} \tag{II.11}$$

Here, since the width (in the x-direction) of the sheet is large enough, no generality is lost by setting the position of crack tip to $\chi = 0$ with $\tau = 0$: the crack exists in the region $\chi < 0$ and is absent in the region $\chi \ge 0$.

We give the boundary conditions for the differential equations (II.11) as follows: the conditions at remote edges, (see Eq. (II.2))

$$\begin{cases} f(-\infty) = 0 & \text{rear side} \\ f(+\infty) = \varepsilon & \text{front side} \end{cases} \tag{II.12}$$

and the matching conditions at the crack tip for the strain field $\mathcal{E}$,

$$\begin{cases} f(-0) = f(+0) \equiv f(0) \\ f'(-0) = f'(+0). \end{cases} \tag{II.13}$$

As previously mentioned, we remove the short spring (located in the center in the y-direction with natural length l, see Fig. S2) when $\mathcal{E}^{(l)}(t) \geq \varepsilon_c$. We rewrite this inequality as

$$\mathcal{E}(\tau, \chi) = f(\chi - \nu\tau) \leq f_c \equiv \frac{N\varepsilon - \varepsilon_c}{N-1}, \tag{II.14}$$

by use of Eq. (II.1). Therefore, $f(0)$ satisfies (i) $f(0) > f_c$ for $\nu = 0$ and (ii) $f(0) = f_c$ for $0 < \nu < \infty$.

We solve the ordinary differential equation (II.11) under the boundary conditions (II.12) and (II.13). First, we solve the differential equation for $\chi < 0$. Substituting the form $f(\chi) = Ce^{-\chi/\xi}$ into Eq. (II.11), we obtain the characteristic equation (quadratic equation for ξ),

$$g_N(\xi) = \xi^2 + \nu\xi - N = 0, \tag{II.15}$$

and the solutions,

$$\xi_{N,\pm} = \left(-\nu \pm \sqrt{\nu^2 + 4N}\right)/2. \tag{II.16}$$

From the boundary conditions (II.12) and (II.13), only the solution $\xi_{N,-}$ is relevant: $f(\chi) = f(0)\exp(-\chi/|\xi_{N,-}|)$. Second, we solve the differential equation for $\chi \geq 0$. Substituting the form $f(\chi) - \varepsilon = Ce^{-\chi/\xi}$ into Eq. (II.11), we obtain the characteristic equation, $g_1(\xi) = \xi^2 + \nu\xi - 1 = 0$, and the solutions, $\xi_{1,\pm} = \left(-\nu \pm \sqrt{\nu^2 + 4}\right)/2$. From the boundary conditions (II.12) and (II.13), only the solution $\xi_{1,+}$ is relevant: $f(\chi) = \varepsilon - [\varepsilon - f(0)]\exp(-\chi/\xi_{1,+})$. Finally, we rewrite the matching condition, $f'(-0) = f'(+0)$ as

$$\frac{f(0)}{|\xi_{N,-}|} = \frac{\varepsilon - f(0)}{\xi_{1,+}}. \tag{II.17}$$

In the following, we determine $f(\chi)$ from Eq. (II.17) in the cases of $\nu = 0$ and $0 < \nu < \infty$.

In the static case ($\nu = 0$), we can rewrite Eq. (II.17) as $f(0) = \frac{\sqrt{N}}{\sqrt{N}+1}\varepsilon$, by using $|\xi_{N,-}| = \sqrt{N}$ and $\xi_{1,+} = 1$ from Eq. (II.16). This gives the following solution:

$$\mathcal{E}(\tau, \chi) = f(\chi) = \begin{cases} \dfrac{\varepsilon\sqrt{N}}{\sqrt{N}+1} e^{\chi/\sqrt{N}} & \text{for } \chi < 0 \quad \text{(rear side)} \\[2ex] \varepsilon - \dfrac{\varepsilon}{\sqrt{N}+1} e^{-\chi} & \text{for } 0 \leq \chi \quad \text{(front side)} \end{cases} \tag{II.18}$$

For $\nu = 0$, the inequality $f(0) > f_c$ yields

$$\tilde{\varepsilon} \equiv \frac{\varepsilon}{\varepsilon_c} < \frac{1}{\sqrt{N}}. \tag{II.19}$$

Note that Eq. (II.19) implies that $\tilde{\varepsilon} > 1/\sqrt{N}$ in the case of crack propagation, $\nu > 0$.

In the case of crack propagation with a constant velocity ν ($0 < \nu < \infty$), since $f(0) = f_c$, the exact relationship between ν and $\tilde{\varepsilon}$ is obtained from Eq. (II.17) as

$$\nu \equiv \frac{V}{V_0} = \frac{N\tilde{\varepsilon}^2 - 1}{\sqrt{\tilde{\varepsilon}(1-\tilde{\varepsilon})(N\tilde{\varepsilon} - 1)}}, \tag{II.20}$$

or equivalently

$$\tilde{\varepsilon} = \frac{\sqrt{\nu^2 + 4N} + \sqrt{\nu^2 + 4}}{\sqrt{\nu^2 + 4N} + N\sqrt{\nu^2 + 4} - (N-1)\nu}. \tag{II.21}$$

The expression (II.20) does not exhibit the velocity jump in the physically relevant range of $\tilde{\varepsilon}$, $1/\sqrt{N} < \tilde{\varepsilon} < 1$. By using Eq. (II.20), we obtain the dynamics of the strain distribution:

$$\mathcal{E}(\tau, \chi) = f(\chi - \nu\tau) = \begin{cases} \dfrac{N\varepsilon - \varepsilon_c}{N-1} \exp\left[\dfrac{\chi - \nu\tau}{|\xi_{N,-}|}\right] & \text{for } \chi - \nu\tau < 0 \quad \text{(rear side)} \\[2ex] \varepsilon - \dfrac{\varepsilon_c - \varepsilon}{N-1} \exp\left[-\dfrac{\chi - \nu\tau}{\xi_{1,+}}\right] & \text{for } 0 \leq \chi - \nu\tau \quad \text{(front side).} \end{cases} \tag{II.22}$$

Here, two healing lengths have been introduced (see Fig. S4c below):

$$|\xi_{N,-}| = \sqrt{\frac{N\tilde{\varepsilon} - 1}{\tilde{\varepsilon}(1 - \tilde{\varepsilon})}}, \qquad \xi_{1,+} = \sqrt{\frac{1 - \tilde{\varepsilon}}{\tilde{\varepsilon}(N\tilde{\varepsilon} - 1)}}. \tag{II.23}$$

Equation (II.20) or (II.21) gives the relation between the velocity V and the initially applied energy density $w = \frac{1}{2}E_0\varepsilon^2$, by virtue of the relation

$$\frac{w}{Nw_0} = \tilde{\varepsilon}^2 \equiv \frac{\varepsilon^2}{\varepsilon_c^2} \quad \text{with} \quad w_0 = \frac{E_0\varepsilon_c^2}{2N}. \tag{II.24}$$

III. MINIMAL MODEL CONSISTING OF ZENER ELEMENTS 1: THE BASIC EQUATIONS AND EXACT SOLUTION

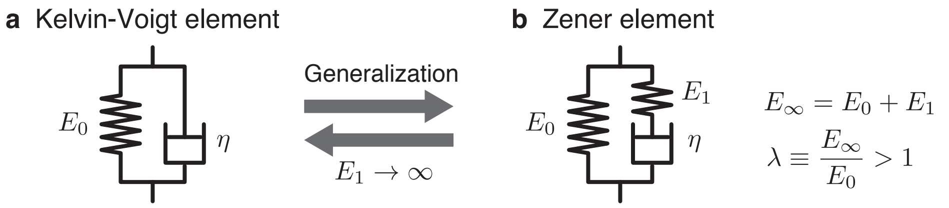

FIG. S3: **(a) Kelvin-Voigt and (b) Zener elements.** The latter reduces to the former in the limit $E_1 \to \infty$.

A. Generalization of viscoelastic interaction: equations of motion in the y-direction

In this section, we generalize the present model by changing the interaction from the one based on Kelvin-Voigt elements to the one on Zener elements (see Fig. S3). A Zener element is a parallel connection of the spring characterized by E_0 (strain $\mathcal{E}(t)$) and a Maxwell element, which is a serial connection of the spring characterized by E_1 (strain $\mathcal{E}_1(t)$) and the dashpot characterized by η (strain $\mathcal{E}_2(t)$). The strains of the parallel components are identical:

$$\mathcal{E}(t) = \mathcal{E}_1(t) + \mathcal{E}_2(t). \tag{III.1}$$

For the serial components in the Maxwell element, the stresses on the spring and dashpot are identical:

$$\eta\frac{\partial}{\partial t}\mathcal{E}_2(t) = E_1\mathcal{E}_1(t). \tag{III.2}$$

Note here that a Zener element reduces to a Kelvin-Voigt element in the limit $E_1 \to \infty$, in which $\mathcal{E}_1(t) = 0$. The generalization of the equations of motion in the y-direction is achieved by the following replacements:

$$\begin{cases} \dfrac{F_{\text{up}}}{ah} + \dfrac{F_{\text{up}}^{(\eta)}}{ah} = E_0\mathcal{E} + \eta\dfrac{\partial}{\partial t}\mathcal{E} & \to \quad E_0\mathcal{E} + \eta\dfrac{\partial}{\partial t}\mathcal{E}_2 \\ \dfrac{F_{\text{up}}}{ah} + \dfrac{F_{\text{up}}^{(\eta)}}{ah} - \dfrac{F_{\text{down}}}{ah} - \dfrac{F_{\text{down}}^{(\eta)}}{ah} = \dfrac{L}{l}E_0\left(\mathcal{E} - \varepsilon\right) + \dfrac{L}{l}\eta\dfrac{\partial}{\partial t}\mathcal{E} & \to \quad \dfrac{L}{l}E_0\left(\mathcal{E} - \varepsilon\right) + \dfrac{L}{l}\eta\dfrac{\partial}{\partial t}\mathcal{E}_2. \end{cases} \tag{III.3}$$

With this replacement, Eq. (II.5) in the continuum limit in the x-direction, $a \to 0$, is given as

$$\begin{cases} 0 = E_0\mathcal{E} + \eta\dfrac{\partial}{\partial t}\mathcal{E}_2 - \dfrac{1}{2}\mu l(L - l)\dfrac{\partial^2}{\partial x^2}\mathcal{E} & \text{rear side} \\ 0 = \dfrac{L}{l}E_0\left(\mathcal{E} - \varepsilon\right) + \dfrac{L}{l}\eta\dfrac{\partial}{\partial t}\mathcal{E}_2 - \dfrac{1}{2}\mu l(L - l)\dfrac{\partial^2}{\partial x^2}\mathcal{E} & \text{front side} \end{cases} \tag{III.4}$$

As in the case of the Kelvin-Voigt interaction, we use the dimensionless parameters, $\tau = t/t_0$, $\chi = x/x_0$, and $N \equiv L/l$, where $t_0 \equiv \eta/E_0$, and $x_0 \equiv l\sqrt{(L-l)\mu/(2LE_0)}$. Then, we rewrite the above equations of motion as

$$\begin{cases} 0 = \mathcal{E} + \dot{\mathcal{E}}_2 - N\mathcal{E}'' & \text{rear side} \\ 0 = (\mathcal{E} - \varepsilon) + \dot{\mathcal{E}}_2 - \mathcal{E}'' & \text{front side,} \end{cases} \tag{III.5}$$

where $\dot{\mathcal{E}}_2 \equiv \frac{\partial}{\partial \tau}\mathcal{E}_2(\tau,\chi)$ and $\mathcal{E}'' \equiv \frac{\partial^2}{\partial \chi^2}\mathcal{E}(\tau,\chi)$.

We eliminate $\mathcal{E}$ in Eq. (III.5) by using the following relation obtained by substituting Eq. (III.2) into Eq. (III.1),

$$\mathcal{E} = \mathcal{E}_2 + \frac{E_0}{E_1}\dot{\mathcal{E}}_2, \tag{III.6}$$

to have the equations of motion for a single field $\mathcal{E}_2$ in the following form:

$$\begin{cases} 0 = \mathcal{E}_2 + (1+s)\dot{\mathcal{E}}_2 - N\mathcal{E}_2'' - sN\dot{\mathcal{E}}_2'' & \text{rear side} \\ 0 = (\mathcal{E}_2 - \varepsilon) + (1+s)\dot{\mathcal{E}}_2 - \mathcal{E}_2'' - s\dot{\mathcal{E}}_2'' & \text{front side.} \end{cases} \tag{III.7}$$

Here, we have introduced the parameter

$$s \equiv \frac{E_0}{E_1}. \tag{III.8}$$

Equation (III.7) with $\mathcal{E}_2$ replaced by $\mathcal{E}$ also holds. This equation for $\mathcal{E}$ can be obtained either (i) by multiplying the operator $1 + s\frac{\partial}{\partial \tau}$ to Eq. (III.7) and use $(1 + s\frac{\partial}{\partial \tau})\mathcal{E}_2 = \mathcal{E}$ or (ii) by directly eliminating $\mathcal{E}_2$ in Eq. (III.5).

In the main text, we have used λ defined as

$$\lambda \equiv \frac{E_\infty}{E_0} = 1 + \frac{1}{s}, \tag{III.9}$$

instead of s. Although the physical meaning of λ is clearer than that of s, we mainly use s in this section for mathematical simplicity. The parameters s and λ vary in the ranges of $0 < s < \infty$ and $1 < \lambda < \infty$, respectively. Note that the Kelvin-Voigt interaction (finite E_0 and $E_1 = \infty$) corresponds to the limit $s \to 0$ or equivalently $\lambda \to \infty$.

B. Derivation of the exact solution of crack propagation with a constant velocity

In this subsection, we solve Eq. (III.7) in the case of crack propagation with a constant velocity ($0 < V < \infty$). Note that, in the case of $V = 0$, a Zener element reduces to a Kelvin-Voigt element, and we have already solved the model consisting of Kelvin-Voigt elements in Sec. II.

We derive the equations of motion with relevant boundary conditions in the present case, following the manner employed in the Kelvin-Voigt case in Sec. II. We substitute the form $\mathcal{E}_2(\tau,\chi) = f(\chi - \nu\tau)$ into Eq. (III.7) and derive linear ordinary differential equations for $f(\chi)$ for $\chi < 0$ and $0 \le \chi$, where we set the position of the crack tip to $\chi = 0$ with $\tau = 0$. Here, V and $\nu = V/V_0$ are the dimensional and dimensionless velocities, respectively, with $V_0 \equiv x_0/t_0 \simeq lE_0/\eta$ as before (see Eq. (II.10)). The result is given by

$$\begin{cases} 0 = f(\chi) - (1+s)\nu f'(\chi) - Nf''(\chi) + s\nu N f'''(\chi) & \text{for } \chi < 0 \quad \text{(rear side)} \\ 0 = f(\chi) - \varepsilon - (1+s)\nu f'(\chi) - f''(\chi) + s\nu f'''(\chi) & \text{for } 0 \le \chi \quad \text{(front side).} \end{cases} \tag{III.10}$$

To determine the boundary conditions, we substitute $\mathcal{E}_2(\tau,\chi) = f(\chi - \nu\tau)$ into Eq. (III.6) to have $\mathcal{E} = f - s\nu f'$. For $\chi - \nu\tau \to \pm\infty$, we have $\mathcal{E} = f$, since $f' = 0$. For $\chi - \nu\tau = 0$ which corresponds to the crack tip, we assume that the strain distributions $\mathcal{E}$ and $\mathcal{E}_2$ are continuous and differentiable at the crack tip. The values of the functions and their derivatives match at the crack tip: $\mathcal{E}(-0) = \mathcal{E}(+0)$, $\mathcal{E}'(-0) = \mathcal{E}'(+0)$, and $\mathcal{E}_2(-0) = \mathcal{E}_2(+0)$ (we can derive $\mathcal{E}_2'(-0) = \mathcal{E}_2'(+0)$ from the others). In addition, as in the case of the Kelvin-Voigt interaction, we assume that the short spring is absent when $\mathcal{E}^{(d)} \ge \varepsilon_c$, which yields $\mathcal{E}(-0) = \mathcal{E}(+0) = \frac{N\varepsilon - \varepsilon_c}{N-1}$. In summary, we give the appropriate boundary conditions as

$$\begin{cases} f(-0) - s\nu f'(-0) = f(+0) - s\nu f'(+0) = \dfrac{N\varepsilon - \varepsilon_c}{N-1}; & \\ f'(-0) = f'(+0); & f''(-0) = f''(+0); \\ f(-\infty) = 0; & f(+\infty) = \varepsilon. \end{cases} \tag{III.11}$$

We can then establish the exact analytical relation between the initially applied strain ε and the normalized velocity of crack propagation $\nu \equiv V/V_0$ as in the following theorem:

Theorem 1 (Relation between initially applied strain and crack-propagation velocity).

If Eqs. (III.10) and (III.11) hold, then

$$\tilde{\varepsilon} \equiv \frac{\varepsilon}{\varepsilon_c} = \frac{\nu\left(1+s+\frac{sN}{\xi_1\xi_N}\right)+\xi_1+\xi_N}{\nu\left(1+sN+\frac{sN}{\xi_1\xi_N}\right)+N\xi_1+\xi_N} = \frac{\frac{\nu}{\lambda-1}\left(\frac{N}{\xi_1\xi_N}+\lambda\right)+\xi_1+\xi_N}{\frac{\nu}{\lambda-1}\left(N+\frac{N}{\xi_1\xi_N}+\lambda-1\right)+N\xi_1+\xi_N}, \tag{III.12}$$

where

$$\xi_N = \frac{1}{6}\left[2^{2/3}\sqrt[3]{3\sqrt{3}\sqrt{-N\left(4N^2+N(4(5-2s)s+1)\nu^2+4s(s+1)^3\nu^4\right)}+9N(2s-1)\nu-2(s+1)^3\nu^3}\right.$$
$$\left.+\frac{2\sqrt[3]{2}\left(3N+(s+1)^2\nu^2\right)}{\sqrt[3]{3\sqrt{3}\sqrt{-N\left(4N^2+N(4(5-2s)s+1)\nu^2+4s(s+1)^3\nu^4\right)}+9N(2s-1)\nu-2(s+1)^3\nu^3}}-2(s+1)\nu\right]. \tag{III.13}$$

We note that ξ_N is the positive solution of the cubic equation

$$g_N(\xi) \equiv \xi^3+(1+s)\nu\xi^2-N\xi-s\nu N=0. \tag{III.14}$$

In the limit $s \to 0$, $g_N(\xi)=0$ reduces to the quadratic equation (II.15), which appears in the case of the Kelvin-Voigt interaction. In this limit, Eqs. (III.12) and (III.13) reduce to Eq. (II.21) and $\xi_N = \left(\sqrt{\nu^2+4N}-\nu\right)/2$, respectively. The uniqueness of ξ_N is ensured by the following lemma.

Lemma 1. *Let s, ν, and N are positive real numbers. Then, $g_N(\xi)=0$ has one positive and two negative real solutions for $-\infty<\xi<\infty$.*

We prove Theorem 1 with the aid of Lemma 1 (we give the proof of Lemma 1 at the end of this subsection).

Proof of Theorem 1. First, we solve the differential equation for $\chi<0$ (the first equation of Eqs. (III.10)). Substituting the form $f(\chi)=Ce^{-\chi/\xi}$ into the equation, we obtain the characteristic equation (III.14). According to Lemma 1, we can set the three solutions, $\xi_{N,1}$, $\xi_{N,2}$, and ξ_N, of the cubic equation $g_N(\xi)=0$ to satisfy the relation $\xi_{N,1}<\xi_{N,2}<0<\xi_N$. To satisfy the boundary conditions at $\xi\to-\infty$, the solution of the differential equation for $\chi<0$ has the form $f(\chi)=\sum_{i=1}^{2}C_ie^{-\chi/\xi_{N,i}}$, where C_1 and C_2 will be determined later by using the boundary conditions at the crack tip, i.e., $\chi=0$.

Second, we solve the differential equation for $\chi\geq 0$. Substituting the form $f(\chi)-\varepsilon=Ce^{-\chi/\xi}$ into the equation, we obtain the characteristic equation,

$$g_1(\xi)=\xi^3+(1+s)\nu\xi^2-\xi-s\nu=0. \tag{III.15}$$

Note that $g_1(\xi)$ is identical to $g_N(\xi)$ with $N=1$. According to Lemma 1, $g_1(\xi)=0$ has one positive and two negative real solutions, and we denote the positive solution as ξ_1. To satisfy the boundary conditions at $\xi\to\infty$, the solution of the differential equation for $\chi>0$ has the form $f(\chi)=\varepsilon-C_0e^{-\chi/\xi_1}$, where we can determine C_0 by using the boundary condition at $\chi=0$:

$$\frac{N\varepsilon-\varepsilon_c}{N-1}=f(+0)-s\nu f'(+0)=\varepsilon-C_0\left(1+\frac{s\nu}{\xi_1}\right). \tag{III.16}$$

Finally, we determine the relation between $\tilde{\varepsilon}$ and ν, by eliminating C_0, C_1, and C_2 from the boundary conditions,

TABLE I: The behaviors of $g_N(\xi)$ as a function of ξ (left) and $g_N(\alpha_-)$ as a function of N (right).

ξ		α_-		0		α_+	
$\frac{\partial}{\partial\xi}g_N(\xi)$	$+$	0	$-$	$-$	$-$	0	$+$
$g_N(\xi)$	$\nearrow$	$g_N(\alpha_-)$	$\searrow$	$-s\nu N$	$\searrow$	$g_N(\alpha_+)$	$\nearrow$

N		$s(s-2)\nu^2$	
$\frac{\partial}{\partial N}g_N(\alpha_-)$	$-$	0	$+$
$g_N(\alpha_-)$	$\searrow$	$s^2\nu^3$	$\nearrow$

$f(-0)=f(+0)$, $f'(-0)=f'(+0)$, and $f''(-0)=f''(+0)$, which can be recast into the following forms:

$$\begin{cases}\varepsilon - C_0 = C_1 + C_2\\ \xi_1^{-1}C_0 = -\xi_{N,1}^{-1}C_1 - \xi_{N,2}^{-1}C_2\\ -\xi_1^{-2}C_0 = \xi_{N,1}^{-2}C_1 + \xi_{N,2}^{-2}C_2.\end{cases} \tag{III.17}$$

Introducing the parameters $D_i \equiv C_i/C_0$ and $\gamma_i \equiv |\xi_1/\xi_{N,i}| = -\xi_1/\xi_{N,i}$, Eq. (III.17) is written as

$$\begin{cases}D_1 + D_2 = \dfrac{\varepsilon}{C_0} - 1 = \left(1+\dfrac{s\nu}{\xi_1}\right)\dfrac{N-1}{\tilde{\varepsilon}^{-1}-1} - 1\\ \gamma_1 D_1 + \gamma_2 D_2 = 1\\ \gamma_1^2 D_1 + \gamma_2^2 D_2 = -1.\end{cases} \tag{III.18}$$

From the second and third expressions of Eq. (III.18), we obtain $D_1 = \frac{\gamma_2+1}{\gamma_1(\gamma_2-\gamma_1)}$ and $D_2 = \frac{-1-\gamma_1}{\gamma_2(\gamma_2-\gamma_1)}$, which leads

$$D_1 + D_2 = \frac{1}{\gamma_1} + \frac{1}{\gamma_2} + \frac{1}{\gamma_1\gamma_2}. \tag{III.19}$$

Here, we note that $\gamma_1 < \gamma_2$. According to Vieta's Formulae (which is for a polynomial equation and relates the coefficients of the polynomial with sums and products of the roots), the characteristic equation $g_N(\xi) = 0$ gives $\xi_{N,1} + \xi_{N,2} + \xi_N = -(1+s)\nu$ and $\xi_{N,1}\xi_{N,2}\xi_N = s\nu N$. These two relations are rewritten as

$$\begin{cases}\dfrac{1}{\gamma_1} + \dfrac{1}{\gamma_2} = -\dfrac{\xi_{N,1}}{\xi_1} - \dfrac{\xi_{N,2}}{\xi_1} = \dfrac{1}{\xi_1}\left[(1+s)\nu + \xi_N\right]\\ \dfrac{1}{\gamma_1\gamma_2} = \dfrac{\xi_{N,1}\xi_{N,2}}{\xi_1^2} = \dfrac{s\nu N}{\xi_1^2\xi_N}.\end{cases} \tag{III.20}$$

Substituting Eqs. (III.20) into Eq. (III.19), we have

$$D_1 + D_2 = \frac{1}{\xi_1}\left[(1+s)\nu + \xi_N\right] + \frac{s\nu N}{\xi_1^2\xi_N}. \tag{III.21}$$

Combining this with the first relation in Eq. (III.18), we have Eq. (III.12). Thus, Theorem 1 is proved. □

Proof of Lemma 1. We express the equation $\frac{\partial}{\partial\xi}g_N(\xi) = 0$ as $3\xi^2 + 2(1+s)\nu\xi - N = 0$ and denote its two solutions as $\alpha_\pm = \left[-(1+s)\nu \pm \sqrt{\nu^2(1+s)^2 + 3N}\right]/3$, where $\alpha_- < 0 < \alpha_+$. The behavior of $g_N(\xi)$ as a function of ξ is summarized in Tab. I; for $\alpha_- \le \xi \le \alpha_+$, the function $g_N(\xi)$ is monotonically decreasing. Then, $g_N(\alpha_+) < g_N(0) = -s\nu N < 0$. If $g_N(\alpha_-) > 0$, $g_N(\xi) = 0$ has one positive and two negative real solutions. Thus, in the following, we show $g_N(\alpha_-) > 0$.

To show $g_N(\alpha_-) > 0$, we calculate $g_N(\alpha_-)$ and its derivative as follows:

$$g_N(\alpha_-) = \frac{2}{27}\left[3N + (s+1)^2\nu^2\right]^{3/2} + \frac{N\nu}{3}(1-2s) + \frac{2}{27}(s+1)^3\nu^3, \tag{III.22}$$

$$\frac{\partial}{\partial N}g_N(\alpha_-) = \frac{1}{3}\left[3N + (s+1)^2\nu^2\right]^{1/2} + \frac{\nu}{3}(1-2s), \tag{III.23}$$

$$\frac{\partial^2}{\partial N^2}g_N(\alpha_-) = \frac{1}{2}\left[3N + (s+1)^2\nu^2\right]^{-1/2}. \tag{III.24}$$

Since s, ν, and N are positive real numbers, $\frac{\partial^2}{\partial N^2}g_N(\alpha_-) > 0$, which means that $\frac{\partial}{\partial N}g_N(\alpha_-)$ is a monotonically increasing function. Now, $\frac{\partial}{\partial N}g_N(\alpha_-) = 0$ at $N = s(s-2)\nu^2$, at which $g_N(\alpha_-)$ takes the minimum value as a function of N. Since $g_N(\alpha_-)|_{N=s(s-2)\nu^2} = s^2\nu^3$ is positive, $g_N(\alpha_-) > 0$ for any N. Thus, Lemma 1 is proved. □

IV. MINIMAL MODEL CONSISTING OF ZENER ELEMENTS 2: LOW- AND HIGH-VELOCITY REGIMES AND VELOCITY JUMP

In this section, we use Eq. (III.12) to investigate the dependences of the initially applied energy density w on the velocity $\nu \equiv V/V_0$ in low- and high-velocity regimes, and derive the existence condition of the velocity jump. Note that Eq. (III.12) gives the relation between the initially applied strain ε and ν and that ε is related to w simply as $w = \tilde{\varepsilon}^2 N w_0$ (see Eq. (II.24)). In the following, the present model will be analyzed for arbitrary positive real number $s \equiv E_0/E_1 > 0$, i.e., for $\lambda \equiv E_\infty/E_0 = 1 + 1/s > 1$. Note that the relations derived below are further simplified for elastomers, for which the relation $1 \ll \lambda \ll N$ is valid (typically $\lambda \simeq 10^2$–10^3 and $N \simeq 10^6$–10^9).

A. Low- and high-velocity regimes

To obtain the asymptotic forms of the initially applied energy w in the low- and high-velocity regimes, we evaluate a solution of the characteristic equation (III.14). We rewrite this equation as

$$G(\Xi) \equiv \frac{g_N(\xi)}{N^{3/2}} = \Xi^3 + \frac{\lambda \upsilon}{\lambda - 1}\Xi^2 - \Xi - \frac{\upsilon}{\lambda - 1} = 0, \tag{IV.1}$$

where $\Xi \equiv \xi/\sqrt{N}$ and $\upsilon \equiv \nu/\sqrt{N}$. We denote the positive real solution of Eq. (IV.1) as $\Xi_+ \equiv \xi_N/\sqrt{N}$, where ξ_N is the positive real solution of Eq. (III.14). We note that Ξ_+ is independent of N and depends only on υ $(0 < \upsilon < \infty)$ and s, as seen from Eq. (IV.1). Then, we obtain the relation between ξ_N and ξ_1: $\Xi_+(\upsilon) = \xi_N(\nu/\sqrt{N})/\sqrt{N} = \xi_1(\nu)$. By evaluating the asymptomatic forms of Ξ_+ in the low- and high-velocity regimes, we have the following lemma:

Lemma 2. *If $0 < \upsilon \equiv \nu/\sqrt{N} < \infty$ and $1 < \lambda < \infty$, then the positive real solution ξ_N of the characteristic equation (III.14), $g_N(\xi) = 0$, has the following asymptotic forms:*

$$\frac{1}{\sqrt{N}}\xi_N(\nu) \equiv \Xi_+(\upsilon) = \begin{cases} 1 - \dfrac{\upsilon}{2} + O\left(\upsilon^2\right) & (\upsilon \to 0) \\ \dfrac{1}{\sqrt{\lambda}} + \dfrac{(\lambda-1)^2}{2\upsilon\lambda^2} + O\left(\dfrac{1}{\upsilon^2}\right) & (\upsilon \to \infty). \end{cases} \tag{IV.2}$$

Note here that (i) if $\nu \equiv V/V_0 \ll \sqrt{N}$, then $\xi_N(\nu) \simeq \sqrt{N}$; and (ii) if $\nu \gg \sqrt{N}(\lambda-1)^2/\lambda^{3/2}$, then $\xi_N(\nu) \simeq \sqrt{N/\lambda}$.

Proof of Lemma 2. We evaluate the asymptomatic form of Ξ_+ in the vicinity of $\upsilon = 0$ (i.e., in the low-velocity regime). Because of the asymptotic behavior, $g_N(\xi)/N^{3/2} \xrightarrow[\upsilon \to 0]{} \Xi^3 - \Xi$, we can express Eq. (IV.1) as $\Xi^3 - \Xi = 0$, which gives the positive real solution, $\Xi = 1$. To evaluate the next order, we introduce the perturbation parameter ϵ_0. Substituting $\Xi = 1 + \epsilon_0$ into Eq. (IV.1), we have

$$2\epsilon_0 + \upsilon + O(\epsilon_0^2, \epsilon_0\upsilon) = 0, \tag{IV.3}$$

whose solution is $\epsilon_0 = -\upsilon/2 + O(\upsilon^2)$. Therefore, we have the expression, $\Xi_+ = 1 - \upsilon/2 + O(\upsilon^2)$.

Similarly, we evaluate the asymptomatic form of Ξ_+ in the vicinity of $1/\upsilon = 0$ (i.e., in the high-velocity regime, $\upsilon \gg 1$). Because of the asymptotic behavior, $g_N(\xi) \cdot (\lambda - 1)/(\upsilon N^{3/2}) \xrightarrow[\upsilon \to \infty]{} \lambda\Xi^2 - 1$, Eq. (IV.1) can be expressed as $\lambda\Xi^2 - 1 = 0$, which gives the positive real solution, $\Xi = 1/\sqrt{\lambda}$. Introducing the perturbation parameter ϵ_∞, and substituting $\Xi = 1/\sqrt{\lambda} + \epsilon_\infty$ into Eq. (IV.1), we have

$$\frac{2\sqrt{\lambda}}{\lambda - 1}\upsilon\epsilon_\infty - \frac{\lambda - 1}{\lambda^{3/2}} - \frac{\lambda - 3}{\lambda}\epsilon_\infty + \frac{\lambda}{\lambda - 1}\upsilon\epsilon_\infty^2 + \frac{3}{\sqrt{\lambda}}\epsilon_\infty^2 + \epsilon_\infty^3 = 0. \tag{IV.4}$$

Since $1 < \lambda < \infty$, $|\epsilon_\infty| \ll 1$, and $\upsilon^{-1} \ll 1$, the first and second terms on the left-hand side of Eq. (IV.4) are the leading-order terms. Thus, the solution of Eq. (IV.4) is $\epsilon_\infty = \frac{1}{2\upsilon}\left(\frac{\lambda-1}{\lambda}\right)^2 + O\left(\upsilon^{-2}\right)$. Therefore, we have the expression, $\Xi_+ = \frac{1}{\sqrt{\lambda}} + \frac{1}{2\upsilon}\left(\frac{\lambda-1}{\lambda}\right)^2 + O\left(\upsilon^{-2}\right)$. □

From Theorem 1, with the aid of Lemma 2, we can evaluate $\tilde{\varepsilon}(\nu)$ in the vicinity of $\nu = 0$ and that of $\nu = \infty$. In the vicinity of $\nu = 0$, $\xi_N(\nu) = \sqrt{N} - \frac{\nu}{2} + O(\nu^2)$ and $\xi_1(\nu) = 1 - \frac{\nu}{2} + O(\nu^2)$. Then, together with Eq. (III.12), we can evaluate $\tilde{\varepsilon}(\nu)$. Similarly, in the vicinity of $\nu = \infty$, $\xi_N(\nu) = \sqrt{\frac{N}{\lambda}} + \frac{N(\lambda-1)^2}{2\nu\lambda^2} + O\left(\frac{1}{\nu^2}\right)$ and $\xi_1(\nu) = \frac{1}{\sqrt{\lambda}} + \frac{(\lambda-1)^2}{2\nu\lambda^2} + O\left(\frac{1}{\nu^2}\right)$. Then, together with Eq. (III.12) we can evaluate $\tilde{\varepsilon}(\nu)$. We summarize the result in the following theorem.

Theorem 2 (Asymptotic forms in low- and high-velocity regimes).

If $\lambda > 1$ *and* $N > 1$*, then*

$$\tilde{\varepsilon}(\nu) = \frac{1}{\sqrt{N}} + \frac{\sqrt{N}-1}{2N}\nu + O\left(\nu^2\right) \qquad (\nu \to 0), \tag{IV.5}$$

and

$$\tilde{\varepsilon}(\nu) = \frac{\lambda}{\sqrt{N}+\lambda-1} - \frac{(\lambda-1)^2\left(\sqrt{N}-1\right)\left(\sqrt{N}+2\right)}{2\nu\sqrt{\lambda}\left(\sqrt{N}+\lambda-1\right)^2} + O\left(\frac{1}{\nu^2}\right) \qquad (\nu \to \infty). \tag{IV.6}$$

Equation (IV.5) enables us to evaluate the relation between the initially applied energy density w and the crack-propagation velocity V in the low-velocity regime (by virtue of the relation $w = \tilde{\varepsilon}^2 N w_0$, see Eq. (II.24)). From Eq. (IV.5), we have $\lim_{\nu\to 0}\tilde{\varepsilon}(\nu) = 1/\sqrt{N}$, or equivalently

$$\lim_{\nu\to 0} w(\nu) = w_0. \tag{IV.7}$$

Comparing the first and second terms on the right-hand side of Eq. (IV.5), we can estimate the range of ν in which $w(\nu) \simeq w_0$ holds: if $1/\sqrt{N} \gg \nu(\sqrt{N}-1)/N$, i.e.,

$$\nu \equiv \frac{V}{V_0} \ll \frac{\sqrt{N}}{\sqrt{N}-1} \tag{IV.8}$$

is satisfied, then $w(\nu) \simeq w_0$ (Here, we have omitted the factor 2 in the second term on the right-hand side of Eq. (IV.5)). When $\sqrt{N} \gg 1$, the condition (IV.8) is simplified as

$$V \ll V_0. \tag{IV.9}$$

Similarly, Eq. (IV.6) enables us to evaluate the w-V relation in the high-velocity regime. From the first term on the right-hand side of Eq. (IV.6), we have $\lim_{\nu\to\infty}\tilde{\varepsilon}(\nu) = \lambda/(\sqrt{N}+\lambda-1)$, or equivalently

$$\lim_{\nu\to\infty} w(\nu) = \frac{\lambda^2 N w_0}{\left(\sqrt{N}+\lambda-1\right)^2}. \tag{IV.10}$$

We can estimate the range of ν in which $w(\nu) \simeq \lambda^2 N w_0/(\sqrt{N}+\lambda-1)^2$ holds, following the manner we employed in the low-velocity case. However, we should pay attention to the fact that Eq. (IV.6) is insufficient for the estimation in the case of (unrealistic) large λ. In fact, for example, in the Kelvin-Voigt limit ($\lambda \to \infty$) the second term on the right-hand side of Eq. (IV.6) goes to zero. Thus, in this discussion, we assume $\lambda \lesssim N$, which includes the case of typical viscoelastic materials ($1 \ll \lambda \ll N$). Comparing the first and second terms on the right-hand side of Eq. (IV.6), we have the range of ν in which $w(\nu) \simeq \lambda^2 N w_0/(\sqrt{N}+\lambda-1)^2$ holds: if the crack propagation velocity is sufficiently high, i.e.,

$$\nu \gg \frac{\left(\sqrt{N}+2\right)\left(\sqrt{N}-1\right)(\lambda-1)^2}{\left(\sqrt{N}+\lambda-1\right)\lambda^{3/2}} \tag{IV.11}$$

is satisfied, then $w(\nu) \simeq \lambda^2 N w_0/(\sqrt{N}+\lambda-1)^2$. In the case of $\sqrt{N} \gg 1$ and $\lambda \gg 1$, which includes the case of typical viscoelastic materials, the above statement can be simplified, i.e., if

$$\nu \gg \frac{\lambda^{1/2} N}{\sqrt{N}+\lambda} \tag{IV.12}$$

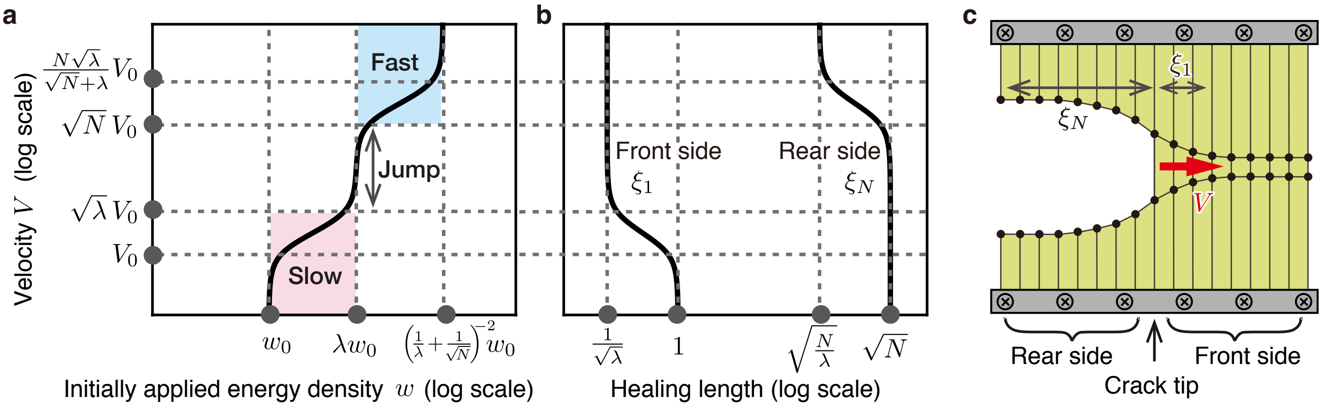

FIG. S4: **Representative plots for typical elastomers ($\lambda = 10^3$, $N = 10^9$). a,** w vs. V; the velocity jump appears because the condition $\lambda \ll N$ is satisfied. **b,** ξ_1 and ξ_N vs. V. **c,** Schematics of the healing lengths ξ_1 and ξ_N, that is, distances over which the disturbance of the stress and strain distributions recover to the remote values (see Eq. (II.22)). As seen in (b), these healing lengths play the roles of order parameters in the context of conventional phase transitions such as superconductors and Bose-Einstein condensations [S2]. In other words, the four velocity scales in plot (a) correspond to crossover points for ξ_1 and ξ_N shown in (b).

is satisfied, then $\tilde{\varepsilon}(\nu) \simeq \frac{\lambda}{\sqrt{N}+\lambda}$, or equivalently

$$w(\nu) \simeq \left(\frac{1}{\sqrt{N}} + \frac{1}{\lambda}\right)^{-2} w_0. \tag{IV.13}$$

We summarize characteristic scales derived from Lemma 2 and Theorem 2 for $1 \ll \lambda \ll N$ in Fig. S4, together with the scales associated with the jump derived in the next subsection.

B. Existence condition of the velocity jump

As illustrated in Fig. S4a, Eq. (III.12) guarantees the existence of the velocity jump under an appropriate condition. In this subsection, we rigorously derive the existence condition of the velocity jump by analyzing Eq. (III.12). Before we give the rigorous derivation, we roughly derive the existence condition of the velocity jump from Theorem 1 with the aid of Lemma 2.

We evaluate the positive solution of the characteristic equation (III.14) under the condition $(\lambda-1)/\sqrt{\lambda} \ll \nu \ll \sqrt{N}$. According to Lemma 2, the positive solution of the characteristic equation $g_N(\xi) = 0$ is expressed as $\xi \simeq \xi_N^{(0)} \equiv \sqrt{N}$ for $\nu \ll \sqrt{N}$. Similarly, the positive solution of $g_1(\xi) = 0$ is expressed as $\xi \simeq \xi_1^{(\infty)} \equiv 1/\sqrt{\lambda}$ for $(\lambda-1)^2/\lambda^{3/2} < (\lambda-1)/\lambda^{1/2} \ll \nu$. Then, under the condition $(\lambda-1)/\sqrt{\lambda} \ll \nu \ll \sqrt{N}$, Eq. (III.12) can be expressed as

$$\tilde{\varepsilon} \equiv \frac{\varepsilon}{\varepsilon_c} \simeq \frac{\frac{\nu}{\lambda-1}\left(\frac{N}{\xi_1^{(\infty)}\xi_N^{(0)}} + \lambda\right) + \xi_1^{(\infty)} + \xi_N^{(0)}}{\frac{\nu N}{\lambda-1}\left(1 + \frac{1}{\xi_1^{(\infty)}\xi_N^{(0)}}\right) + \nu + N\xi_1^{(\infty)} + \xi_N^{(0)}} = \frac{\frac{\nu}{\lambda-1}\left(\lambda + \sqrt{\lambda N}\right) + \frac{1}{\sqrt{\lambda}} + \sqrt{N}}{\frac{\nu}{\lambda-1}\left(N + \sqrt{\lambda N}\right) + \nu + \frac{N}{\sqrt{\lambda}} + \sqrt{N}}. \tag{IV.14}$$

By noting the relation $\frac{1}{\sqrt{\lambda}} + \sqrt{N} < \sqrt{\lambda} + \sqrt{N} \ll \frac{\nu}{\lambda-1}\left(\lambda + \sqrt{\lambda N}\right)$ for the numerator in Eq. (IV.14) and the relation

$$\frac{N}{\sqrt{\lambda}} + \sqrt{N} \ll \frac{\nu N}{\lambda - 1}\left(1 + \sqrt{\frac{\lambda}{N}}\right) \tag{IV.15}$$

for the denominator in Eq. (IV.14), we obtain

$$\tilde{\varepsilon} \simeq \frac{\sqrt{\lambda N} + \lambda}{N + \sqrt{\lambda N} + \lambda - 1} \simeq \frac{\sqrt{\lambda N} + \lambda}{N + \sqrt{\lambda N}} = \sqrt{\frac{\lambda}{N}}. \tag{IV.16}$$

Therefore, we obtain the following rough estimation for the velocity jump.

If $1 < \lambda < \infty$ and $(\lambda-1)/\sqrt{\lambda} \ll \sqrt{N}$, then the approximate expression $\tilde{\varepsilon}(\nu) \equiv \varepsilon(\nu)/\varepsilon_c \simeq \sqrt{\lambda/N}$ is valid for ν in the range $(\lambda-1)/\sqrt{\lambda} \ll \nu \ll \sqrt{N}$.

Note that $\tilde{\varepsilon}(\nu) \simeq \sqrt{\lambda/N}$ corresponds to $w(\nu) \simeq w_{\rm jump} \equiv \lambda w_0$, which is the position of the velocity jump shown in Fig. S4a.

To complete the proof of the existence of the velocity jump, we clarify in Theorem 3 below the magnitude of the correction for the expression $\tilde{\varepsilon}(\nu) \simeq \sqrt{\frac{\lambda}{N}}$ with using rigorous inequalities. For the preparation to prove Theorem 3, we summarize necessary inequalities in the following lemma:

Lemma 3. *If $0 < \upsilon < \infty$ and $1 < \lambda < \infty$, then the positive real solution $\Xi_+(\upsilon,\lambda)$ of the characteristic equation (IV.1), $G(\Xi) \equiv \Xi^3 + \frac{\lambda\upsilon}{\lambda-1}\Xi^2 - \Xi - \frac{\upsilon}{\lambda-1} = 0$, satisfies the following inequalities:*

$$\frac{1}{1+\upsilon} < \Xi_+(\upsilon,\lambda) < 1, \tag{IV.17}$$

$$1-\frac{\upsilon}{2} < \Xi_+(\upsilon,\lambda) \qquad \text{for} \quad 0<\upsilon<2, \tag{IV.18}$$

$$\frac{1}{\sqrt{\lambda}} < \Xi_+(\upsilon,\lambda) < \frac{1}{\sqrt{\lambda}} + \frac{(\lambda-1)^2}{\upsilon\lambda^2}, \tag{IV.19}$$

$$\sqrt{\lambda} - \frac{(\lambda-1)^2}{2\upsilon\lambda} < \frac{1}{\Xi_+(\upsilon,\lambda)} < \sqrt{\lambda} - \frac{(\lambda-1)^2}{4\upsilon\lambda} \qquad \text{for} \quad \frac{\lambda-1}{\sqrt{\lambda}} < \upsilon. \tag{IV.20}$$

Proof of Lemma 3. We note that (i) $G(\Xi) < 0$ for $0 < \Xi < \Xi_+$ and (ii) $G(\Xi) > 0$ for $\Xi_+ < \Xi$. This is because $G(0) = -\upsilon/(\lambda-1) < 0$ and the equation $G(\Xi) = 0$ has a unique positive real solution Ξ_+ as guaranteed by Lemma 1. In the following, we prove the inequalities in Lemma 3, which means that $G(a) < 0$ or $G(a) > 0$ for positive real numbers a. First, Eq. (IV.17) holds because

$$G\left(\frac{1}{1+\upsilon}\right) = -\frac{\upsilon}{(\upsilon+1)^3} - \frac{\upsilon^2(\upsilon+2)}{(\lambda-1)(\upsilon+1)^2} < 0 \tag{IV.21}$$

and $G(1) = \upsilon > 0$ for $0 < \upsilon$ and $1 < \lambda$. Second, Eq. (IV.18) holds because

$$G\left(1-\frac{\upsilon}{2}\right) = \frac{\upsilon^2}{8(\lambda-1)}\left[\lambda(\upsilon-2)+\upsilon-6\right] < 0 \tag{IV.22}$$

for $0 < \upsilon < 2$ and $1 < \lambda$. Third, Eq. (IV.19) holds because $G\left(\frac{1}{\sqrt{\lambda}}\right) = \frac{1-\lambda}{\lambda^{3/2}} < 0$ for $1 < \lambda < \infty$ and

$$G\left(\frac{1}{\sqrt{\lambda}} + \frac{(\lambda-1)^2}{\upsilon\lambda^2}\right) = \frac{\lambda-1}{\lambda^6\upsilon^3}\left[\lambda^{9/2}\upsilon^3 + \lambda^3(\lambda-1)\upsilon^2 + 3\lambda^{3/2}(\lambda-1)^3\upsilon + (\lambda-1)^5\right] > 0 \tag{IV.23}$$

for $0 < \upsilon$ and $1 < \lambda$. Finally, to prove Eq. (IV.20), we show the following inequalities for Ξ_+:

$$\left[\sqrt{\lambda} - \frac{(\lambda-1)^2}{4\upsilon\lambda}\right]^{-1} < \Xi_+(\upsilon,\lambda) < \left[\sqrt{\lambda} - \frac{(\lambda-1)^2}{2\upsilon\lambda}\right]^{-1} \tag{IV.24}$$

for $1 < \lambda$ and $(\lambda-1)/\sqrt{\lambda} < \upsilon$. To obtain the above lower bound of Ξ_+, we calculate

$$G\left(\left[\sqrt{\lambda} - \frac{(\lambda-1)^2}{4\upsilon\lambda}\right]^{-1}\right) = -\frac{(\lambda-1)\upsilon\,\tilde{G}(\upsilon)}{\left[4\lambda^{3/2}\upsilon - (\lambda-1)^2\right]^3}, \tag{IV.25}$$

where

$$\begin{aligned} \tilde{G}(\upsilon) &\equiv 32\lambda^3\upsilon^2 - 4(5\lambda+3)(\lambda-1)\lambda^{3/2}\upsilon + (\lambda-1)^3(3\lambda+1) \\ &= 32\left[\lambda^{3/2}\upsilon - \frac{1}{16}(5\lambda+3)(\lambda-1)\right]^2 + (\lambda-1)\left(3\lambda^3 - 5\lambda^2 + \frac{3}{8}\lambda + \frac{5}{8}\right). \end{aligned} \tag{IV.26}$$

Since $1 < \lambda$ and $(\lambda-1)/\sqrt{\lambda} < \upsilon$, we have $\lambda^{3/2}\upsilon > \lambda(\lambda-1) > \frac{1}{16}(5\lambda+3)(\lambda-1)$. Thus, $\tilde{G}(\upsilon)$ is a monotonically increasing function of υ and we obtain

$$\tilde{G}(\upsilon) > \tilde{G}\left(\frac{\lambda-1}{\sqrt{\lambda}}\right) = (\lambda-1)^3(15\lambda+1) > 0. \tag{IV.27}$$

Therefore, Eq. (IV.25) is negative. To obtain the above upper bound of Ξ_+, we calculate

$$G\left(\left[\sqrt{\lambda} - \frac{(\lambda-1)^2}{2\upsilon\lambda}\right]^{-1}\right) = \frac{(\lambda-1)^2\upsilon\,\tilde{\tilde{G}}(\upsilon)}{\left[2\lambda^{3/2}\upsilon - (\lambda-1)^2\right]^3}, \tag{IV.28}$$

where $\tilde{\tilde{G}}(\upsilon) \equiv 2(\lambda+3)\lambda^{3/2}\upsilon - (\lambda-1)^2(\lambda+1)$. Since $1 < \lambda$ and $(\lambda-1)/\sqrt{\lambda} < \upsilon$, we have

$$\tilde{\tilde{G}}(\upsilon) > 2(\lambda-1)\lambda(\lambda+3) - (\lambda-1)^2(\lambda+1) = (\lambda-1)(\lambda^2+6\lambda+1) > 0 \tag{IV.29}$$

and Eq. (IV.28) is positive. Therefore, Eq. (IV.20) is proved. □

Theorem 3 (Existence condition of the velocity jump).

If $1 < \lambda < \infty$, $1 < N < \infty$, *and*

$$\frac{\lambda-1}{\sqrt{\lambda}} < \nu < \sqrt{N}, \tag{IV.30}$$

then the initially applied strain $\varepsilon = \varepsilon(\nu, \lambda, N)$ *is bounded as follows:*

$$\sqrt{\frac{\lambda}{N}}\left(1 - \frac{\lambda-1}{\nu\sqrt{\lambda}} - \frac{\nu\sqrt{\lambda}}{N}\right) < \tilde{\varepsilon} \equiv \frac{\varepsilon}{\varepsilon_c} < \sqrt{\frac{\lambda}{N}}\left(1 + \frac{\nu}{\sqrt{N}}\right). \tag{IV.31}$$

Thus, the approximate expression

$$\tilde{\varepsilon} \simeq \sqrt{\frac{\lambda}{N}} \tag{IV.32}$$

is valid in the range of ν,

$$\frac{\lambda-1}{\sqrt{\lambda}} \ll \nu \ll \sqrt{N}. \tag{IV.33}$$

Here, to derive Eq. (IV.32), we use $\lim_{\nu/\sqrt{N}\to 0} \nu\sqrt{\lambda}/N = 0$ in Eq. (IV.31). This equality is derived as follows. If $1 < \lambda < \infty$, $1 < N < \infty$, and $(\lambda-1)/\sqrt{\lambda} < \nu < \sqrt{N}$, then $0 < \sqrt{\lambda/N} < (1+\sqrt{5})/2$. Thus, $0 < \nu\sqrt{\lambda}/N < (1+\sqrt{5})\nu/(2\sqrt{N}) \xrightarrow[\nu \ll \sqrt{N}]{} 0$.

We have two remarks for Theorem 3: (i) the present model consisting of Kelvin-Voigt elements (which corresponds to $\lambda \to \infty$) never satisfies the existence condition of velocity jump in Eq. (IV.33) because N is finite; (ii) if $\lambda \gg 1$, then the existence condition (IV.33) reduces to

$$\sqrt{\lambda} \ll \nu \ll \sqrt{N}. \tag{IV.34}$$

Proof of Theorem 3. We evaluate the numerator $\tilde{\varepsilon}^{(\mathrm{nu})}$ and denominator $\tilde{\varepsilon}^{(\mathrm{de})}$ of Eq. (III.12) in the range of ν given in Eq. (IV.30), where

$$\tilde{\varepsilon}^{(\mathrm{nu})} \equiv \frac{1}{\xi_1\xi_N} + \frac{\lambda}{N} + \frac{\lambda-1}{\nu N}\left(\xi_1 + \xi_N\right), \tag{IV.35}$$

$$\tilde{\varepsilon}^{(\mathrm{de})} \equiv 1 + \frac{1}{\xi_1\xi_N} + \frac{\lambda-1}{\nu N}\left(\nu + N\xi_1 + \xi_N\right), \tag{IV.36}$$

with $\tilde{\varepsilon} = \tilde{\varepsilon}^{(\mathrm{nu})}/\tilde{\varepsilon}^{(\mathrm{de})}$. By using Eqs. (IV.17)–(IV.20) with $\Xi_+ \equiv \xi_N/\sqrt{N}$ and $\upsilon \equiv \nu/\sqrt{N}$, we have the following bounds:

$$\sqrt{N} - \frac{\nu}{2} < \xi_N < \sqrt{N} \qquad \text{for} \quad 0 < \nu < 2\sqrt{N}, \tag{IV.37}$$

$$\frac{1}{\sqrt{\lambda}} < \xi_1 < \frac{1}{\sqrt{\lambda}} + \frac{(\lambda-1)^2}{\nu\lambda^2}, \tag{IV.38}$$

$$\sqrt{\frac{\lambda}{N}} - \frac{(\lambda-1)^2}{2\nu\lambda\sqrt{N}} < \frac{1}{\xi_1\xi_N} < \left(\sqrt{\frac{\lambda}{N}} + \frac{\nu\sqrt{\lambda}}{N}\right)\left(1 - \frac{(\lambda-1)^2}{4\nu\lambda^{3/2}}\right) \qquad \text{for} \quad \frac{\lambda-1}{\sqrt{\lambda}} < \nu, \tag{IV.39}$$

where the condition in Eq. (IV.39) is satisfied because $(\lambda-1)^2/\lambda^{3/2} < (\lambda-1)/\lambda^{1/2}$. Here, we note that $\Xi_+ = \xi_1$ and $\upsilon = \nu$ for $N = 1$. By evaluating Eqs. (IV.35) and (IV.36) with these bounds, we have

$$\frac{\lambda^2-1}{2\nu\lambda\sqrt{N}} + \frac{\lambda-1}{\nu N\sqrt{\lambda}} - \frac{\lambda-1}{2N} < \tilde{\varepsilon}^{(\mathrm{nu})} - \sqrt{\frac{\lambda}{N}} - \frac{\lambda}{N} < \frac{\nu\sqrt{\lambda}}{N} + \frac{\lambda-1}{\nu N}\left(\sqrt{N} + \frac{1}{\sqrt{\lambda}} + \frac{(\lambda-1)^2}{\nu\lambda^2}\right), \tag{IV.40}$$

$$\frac{\lambda^2-1}{2\nu\lambda\sqrt{N}} + \frac{\lambda-1}{\nu\sqrt{\lambda}} + \frac{\lambda-1}{2N} < \tilde{\varepsilon}^{(\mathrm{de})} - 1 - \sqrt{\frac{\lambda}{N}} < \frac{\nu\sqrt{\lambda}}{N} + \frac{\lambda-1}{\nu N}\left(\nu + \sqrt{N} + \frac{N}{\sqrt{\lambda}} + \frac{(\lambda-1)^2 N}{\nu\lambda^2}\right). \tag{IV.41}$$

Here, to give the lower bounds in Eqs. (IV.40) and (IV.41), we use

$$\frac{\lambda-1}{\nu\sqrt{N}} - \frac{(\lambda-1)^2}{2\nu\lambda\sqrt{N}} = \frac{\lambda^2-1}{2\nu\lambda\sqrt{N}}. \tag{IV.42}$$

Equations (IV.40) and (IV.41) yield $\tilde{\varepsilon}_{\mathrm{lower}} < \tilde{\varepsilon} < \tilde{\varepsilon}_{\mathrm{upper}}$, where

$$\tilde{\varepsilon}_{\mathrm{lower}} \equiv \frac{\sqrt{\frac{\lambda}{N}} + \frac{\lambda}{N} + \frac{\lambda^2-1}{2\nu\lambda\sqrt{N}} + \frac{\lambda-1}{\nu N\sqrt{\lambda}} - \frac{\lambda-1}{2N}}{1 + \sqrt{\frac{\lambda}{N}} + \frac{\nu\sqrt{\lambda}}{N} + (\lambda-1)\left(\frac{1}{N} + \frac{1}{\nu\sqrt{N}} + \frac{1}{\nu\sqrt{\lambda}} + \frac{(\lambda-1)^2}{\nu^2\lambda^2}\right)}, \tag{IV.43}$$

$$\tilde{\varepsilon}_{\mathrm{upper}} \equiv \frac{\sqrt{\frac{\lambda}{N}} + \frac{\lambda}{N} + \frac{\nu\sqrt{\lambda}}{N} + \frac{\lambda-1}{\nu N}\left(\sqrt{N} + \frac{1}{\sqrt{\lambda}} + \frac{(\lambda-1)^2}{\nu\lambda^2}\right)}{1 + \sqrt{\frac{\lambda}{N}} + \frac{\lambda^2-1}{2\nu\lambda\sqrt{N}} + \frac{\lambda-1}{\nu\sqrt{\lambda}} + \frac{\lambda-1}{2N}}. \tag{IV.44}$$

We evaluate the upper bound $\tilde{\varepsilon}_{\mathrm{upper}}$ as follows.

$$\begin{aligned}
\tilde{\varepsilon}_{\mathrm{upper}} &= \sqrt{\frac{\lambda}{N}} + \frac{\frac{\nu\sqrt{\lambda}}{N} + \frac{(\lambda-1)^3}{\nu^2\lambda^2 N} - \frac{(\lambda-1)^2}{2\nu N\sqrt{\lambda}} - \frac{\lambda-1}{2N}\sqrt{\frac{\lambda}{N}}}{1 + \sqrt{\frac{\lambda}{N}} + \frac{\lambda^2-1}{2\nu\lambda\sqrt{N}} + \frac{\lambda-1}{\nu\sqrt{\lambda}} + \frac{\lambda-1}{2N}} \\
&< \sqrt{\frac{\lambda}{N}} + \frac{\frac{\nu\sqrt{\lambda}}{N} + \frac{(\lambda-1)^3}{\nu^2\lambda^2 N}}{1 + \frac{\lambda-1}{\nu\sqrt{\lambda}}} \\
&= \sqrt{\frac{\lambda}{N}} + \frac{\nu\sqrt{\lambda}}{N} + \frac{\frac{(\lambda-1)^3}{\nu^2\lambda^2 N} - \frac{\lambda-1}{N}}{1 + \frac{\lambda-1}{\nu\sqrt{\lambda}}} \\
&< \sqrt{\frac{\lambda}{N}} + \frac{\nu\sqrt{\lambda}}{N} \\
&= \sqrt{\frac{\lambda}{N}}\left(1 + \frac{\nu}{\sqrt{N}}\right),
\end{aligned} \tag{IV.45}$$

where we have used

$$\frac{(\lambda-1)^3}{\nu^2\lambda^2 N} - \frac{\lambda-1}{N} = \frac{\lambda-1}{N}\left[\frac{(\lambda-1)^2}{\nu^2\lambda^2} - 1\right] < 0. \tag{IV.46}$$

We evaluate the lower bound $\tilde{\varepsilon}_{\text{lower}}$ as follows.

$$\begin{aligned}
\tilde{\varepsilon}_{\text{lower}} &= \sqrt{\frac{\lambda}{N}} + \frac{\frac{\lambda^2-1}{2\nu\lambda\sqrt{N}} + \frac{\lambda-1}{\nu N\sqrt{\lambda}} - \frac{\lambda-1}{2N} - \sqrt{\frac{\lambda}{N}}\left[\frac{\nu\sqrt{\lambda}}{N} + (\lambda-1)\left(\frac{1}{N} + \frac{1}{\nu\sqrt{N}} + \frac{1}{\nu\sqrt{\lambda}} + \frac{(\lambda-1)^2}{\nu^2\lambda^2}\right)\right]}{1 + \sqrt{\frac{\lambda}{N}} + \frac{\nu\sqrt{\lambda}}{N} + (\lambda-1)\left(\frac{1}{N} + \frac{1}{\nu\sqrt{N}} + \frac{1}{\nu\sqrt{\lambda}} + \frac{(\lambda-1)^2}{\nu^2\lambda^2}\right)} \\
&= \sqrt{\frac{\lambda}{N}} - \frac{\lambda-1}{\nu\sqrt{N}} + \frac{\frac{(\lambda-1)\alpha}{\nu\sqrt{N}} - \frac{\nu\lambda}{N\sqrt{N}}}{1 + \sqrt{\frac{\lambda}{N}} + \frac{\nu\sqrt{\lambda}}{N} + (\lambda-1)\left(\frac{1}{N} + \frac{1}{\nu\sqrt{N}} + \frac{1}{\nu\sqrt{\lambda}} + \frac{(\lambda-1)^2}{\nu^2\lambda^2}\right)} \\
&> \sqrt{\frac{\lambda}{N}} - \frac{\lambda-1}{\nu\sqrt{N}} - \frac{\nu\lambda}{N\sqrt{N}} \\
&= \sqrt{\frac{\lambda}{N}}\left(1 - \frac{\lambda-1}{\nu\sqrt{\lambda}} - \frac{\nu\sqrt{\lambda}}{N}\right),
\end{aligned} \tag{IV.47}$$

where we have introduced a positive number α:

$$\alpha = \left(\frac{1}{2} - \frac{\nu}{2\sqrt{N}}\right) + \left(\frac{\lambda-1}{\nu\sqrt{\lambda}} - \frac{(\lambda-1)^2}{\nu\lambda^{3/2}}\right) + \frac{1}{2\lambda} + \frac{1}{\sqrt{\lambda N}} + (\lambda-1)\left(\frac{1}{N} + \frac{1}{\nu\sqrt{N}} + \frac{(\lambda-1)^2}{\nu^2\lambda^2}\right). \tag{IV.48}$$

Thus, Eq. (IV.31) is proved. □

[S1] Cardy, J. *Scaling and renormalization in statistical physics* (Cambridge Univ. press, Cambridge, 1996).

[S2] Leggett, A. J. *Quantum Liquids: Bose condensation and Cooper pairing in condensed-matter systems* (Oxford Univ. Press, Oxford, 2006).
